# Visual Sensory Cortices Causally Contribute to Auditory Word Recognition Following Sensorimotor-Enriched Vocabulary Training

**DOI:** 10.1093/cercor/bhaa240

**Published:** 2020-09-22

**Authors:** Brian Mathias, Leona Sureth, Gesa Hartwigsen, Manuela Macedonia, Katja M Mayer, Katharina von Kriegstein

**Affiliations:** Chair of Cognitive and Clinical Neuroscience, Faculty of Psychology, Technical University Dresden, Dresden 01187, Germany; Research Group Neural Mechanisms of Human Communication, Max Planck Institute for Human Cognitive and Brain Sciences, Leipzig, Germany; Research Group Neural Mechanisms of Human Communication, Max Planck Institute for Human Cognitive and Brain Sciences, Leipzig, Germany; Lise Meitner Research Group Cognition and Plasticity, Max Planck Institute for Human Cognitive and Brain Sciences, Leipzig, Germany; Research Group Neural Mechanisms of Human Communication, Max Planck Institute for Human Cognitive and Brain Sciences, Leipzig, Germany; Institute for Information Engineering, Johannes Kepler University Linz, Linz, Austria; Institute of Psychology, University of Münster, Münster, Germany; Chair of Cognitive and Clinical Neuroscience, Faculty of Psychology, Technical University Dresden, Dresden 01187, Germany; Research Group Neural Mechanisms of Human Communication, Max Planck Institute for Human Cognitive and Brain Sciences, Leipzig, Germany

**Keywords:** biological motion, foreign language learning, gesture, sensorimotor learning, TMS

## Abstract

Despite a rise in the use of “learning by doing” pedagogical methods in praxis, little is known as to how the brain benefits from these methods. Learning by doing strategies that utilize complementary information (“enrichment”) such as gestures have been shown to optimize learning outcomes in several domains including foreign language (L2) training. Here we tested the hypothesis that behavioral benefits of gesture-based enrichment are critically supported by integrity of the biological motion visual cortices (bmSTS). Prior functional neuroimaging work has implicated the visual motion cortices in L2 translation following sensorimotor-enriched training; the current study is the first to investigate the causal relevance of these structures in learning by doing contexts. Using neuronavigated transcranial magnetic stimulation and a gesture-enriched L2 vocabulary learning paradigm, we found that the bmSTS causally contributed to behavioral benefits of gesture-enriched learning. Visual motion cortex integrity benefitted both short- and long-term learning outcomes, as well as the learning of concrete and abstract words. These results adjudicate between opposing predictions of two neuroscientific learning theories: While reactivation-based theories predict no functional role of specialized sensory cortices in vocabulary learning outcomes, the current study supports the predictive coding theory view that these cortices precipitate sensorimotor-based learning benefits.

## Introduction

Foreign language (L2) vocabulary learning by adults is effortful and time-consuming. Words must be relearned often to build up robust memory representations. L2 vocabulary learning typically relies on unisensory materials such as written word lists or audio recordings (Choo et al. 2012). More recent learning-by-doing-based approaches contrast with these techniques. Though initially viewed as unconventional, principles of learning by doing have shifted from the periphery of educational science toward its center over the past few decades. Learning by doing strategies make use of visual and somatosensory information as well as motor information. We therefore in the following refer to learning by doing strategies as “sensorimotor-enriched” learning. Sensorimotor-enriched learning methods boost test performance in science, engineering, mathematics, and L2 learning compared with other learning methods (Freeman et al. 2014; reviewed in Macedonia 2014).

Brain mechanisms underlying enhanced memory for sensorimotor-enriched stimuli remain elusive. It has been suggested that the presence of complementary sensory or motor information during learning induces the formation of both motoric (Hommel et al. 2001) and multisensory (Barsalou 1999) memories, establishing a greater number of routes for successful memory retrieval. The translation of auditorily presented L2 words that have previously been learned using gestures elicits responses within pre−/motor cortices in adults (Mayer et al. 2015). Auditory L2 translation also elicits specialized visual cortical responses: Whereas the biological motion area of the superior temporal sulcus (bmSTS) becomes engaged following gesture-enriched vocabulary learning, the lateral occipital complex (LOC) is engaged following picture-enriched vocabulary learning (Mayer et al. 2015). These neuroimaging results are consistent with studies showing that the presence of complementary sensory information during learning elicits reactivation of specialized sensory brain regions at test (Lahav et al. 2007; Danker and Anderson 2010). The results are also consistent with embodied theories of semantic processing, which propose that word meanings are represented in an experience-dependent network of sensory and motor areas (reviewed in Barsalou 2008; Meteyard et al. 2012; Kiefer and Pulvermüller 2012; Hald et al. 2016; Lambon Ralph et al. 2017).

The reactivation of visual brain regions elicited by stimuli presented in the auditory modality following sensorimotor-enriched learning may be viewed as epiphenomenal, a view taken by reactivation theories of multisensory learning (Nyberg et al. 2000; Wheeler et al. 2000; Fuster 2009). For example, if the taste of a cake triggered the recall of a visual memory, the recollection might reactivate visual areas; reactivation theories, however, assume that those visual responses do not aid in making the sensory experience of the cake’s taste more precise. Within a reactivation-based framework, benefits of sensorimotor enrichment on learning could be relegated to arousal-based effects that are not dependent on representations subserved by sensory brain regions.

Reactivation theories can be seen as a critical counterpart to the notion that sensorimotor networks formed during real-world experience support perception (reviewed in Barsalou 2010; von Kriegstein 2012; Matusz et al. 2017). According to this alternate view, sensory brain responses to previously learned items directly benefit recognition processes by increasing recognition speed and accuracy. The predictive coding theory of multisensory learning (von Kriegstein and Giraud 2006; Mayer et al. 2015; reviewed in von Kriegstein 2012) takes this approach by proposing that sensory and motor cortices build up sensorimotor (e.g., visuomotor) forward models during perception that simulate missing input, which benefits behavioral learning outcomes. The first aim of the current study was to test the opposing predictions of reactivation-based and predictive coding theories of multisensory learning. Adjudicating between these predictions is important because, if sensory representations support multisensory learning benefits, teaching techniques could be optimized to target specific sensory structures that underlie task performance.

The second aim of the current study was to evaluate the functional role of sensory brain responses in learning outcomes over an extended time period (>5 months post training). Human memory is often partitioned into procedural memory, which is anchored in sensory and motor systems and declarative memory for facts and events (Tulving and Madigan 1970; Cohen et al. 1997; Squire and Dede 2015). Though memory for vocabulary has been construed traditionally as a form of declarative memory (Cabeza and Moscovitch 2013), sensorimotor-enriched training may anchor L2 vocabulary representations in procedural memory systems. Procedural memories decay less rapidly than newly learned declarative memories (Mitchell et al. 1990; Tunney 2003) and are less vulnerable to interference following stabilization (reviewed in Robertson et al. 2004). In line with this dissociation, gesture-enriched L2 vocabulary tends to be better remembered than picture-enriched vocabulary several months after L2 training has ended (Mayer et al. 2015). Given the temporal robustness of sensorimotor-enriched learning benefits, we expected visual representations to support learning benefits over extended posttraining durations (>5 months post training), in congruence with the predictive coding theory of multisensory learning.

A potentially limiting factor in the success of sensorimotor-enriched approaches to L2 word learning may arise from word concreteness, that is, the perceptibility of a word’s referent. Perceptibility refers to the extent to which referents can be perceived by the body’s sensory systems (e.g., tangibility and visibility; Paivio et al. 1968; Hoffman 2016). The referent of the concrete noun “ball,” for example, is highly tangible and highly visible and can be iconically represented by using one’s arms to throw an imaginary ball. Referents of other words, such as the abstract noun “mentality,” are less tangible or visible and more difficult to convey using gestures or pictures. Despite these differences, the learning of both concrete and abstract vocabulary has been found to benefit from sensorimotor-enriched learning (Macedonia 2014; Mayer et al. 2015). Given that sensorimotor enrichment can facilitate the learning of both concrete and abstract words, our third aim was to test the hypothesis that visual representations would support posttraining benefits conferred on both word types. Such a finding would be congruent not only with the predictive coding theory of multisensory learning but also with embodied theories of semantics, which propose that an understanding of abstract concepts critically relies on simulations of social, emotional, or sensorimotor aspects of situations (Glenberg and Kaschak 2002; Jamrozik et al. 2016; reviewed in Barsalou 1999; Barsalou and Wiemer-Hastings 2005).

Though functional neuroimaging has contributed much to our understanding of interactions between information arising from distinct sensory modalities (reviewed in James and Stevenson 2012), neuroimaging techniques are limited to demonstrations of correlational rather than causal effects (Ramsey et al. 2010). In the current study, we addressed our three aims by using transcranial magnetic stimulation (TMS) to investigate whether sensory cortices causally contribute to the benefits of sensorimotor-enriched learning. We used a standard gesture-enriched L2 learning paradigm, in which adult learners were trained on novel L2 words over several days. TMS was used to target the bmSTS, a region implicated in the visual perception of biological movements (Grossman et al. 2000). Previous fMRI studies have revealed that this region is engaged during the translation of gesture-enriched L2 vocabulary (Mayer et al. 2015).

## Materials and Methods

### Study Overview and Hypotheses

**Figure 1:**
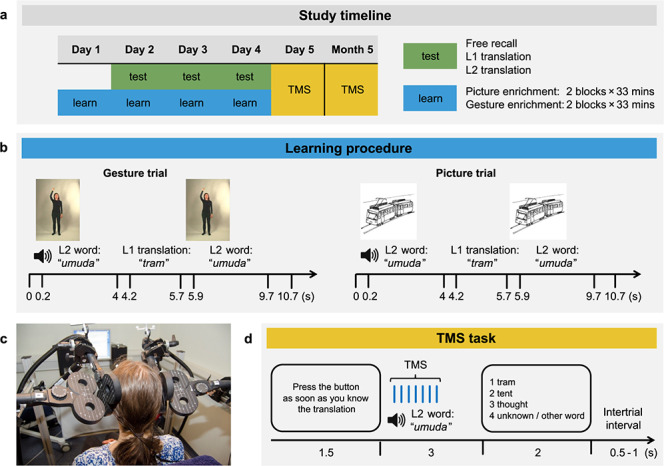
Experimental procedure and design. (*a*) Participants learned L2 vocabulary over four consecutive days (‘learn’) in groups to emulate a classroom setting. Free recall and translation tests (‘test’) were administered on days 2 through 4. TMS sessions occurred during day 5 and month 5. (*b*) In both gesture and picture learning conditions, participants heard an L2 word, followed by the translation in their native language (L1) and a repetition of the L2 word. Videos of iconic gestures and pictures accompanied L2 words in gesture and picture trials, respectively. Participants performed the gesture along with the video during its repetition. (*c*) During both TMS sessions (day 5 and month 5), two TMS coils targeted the bilateral biological motion superior temporal sulcus (bmSTS) using stereotactic neuronavigation based on individual structural brain scans. Two additional coils generated ineffective placebo stimulation (i.e., sham TMS) and were positioned on top of the bmSTS coils at an angle of 90°. (*d*) During each TMS session, participants heard the L2 words that they had learned during the 4-day training and pressed a button as soon as the L1 translation came to their mind (recall task). They then selected the L1 translation by button press from a list of options presented on a screen (multiple-choice task). L1 words were presented in German. Trains of seven TMS pulses at 10 Hz were delivered 50 ms following each L2 word onset. Trials with effective and sham TMS alternated in blocks.

Adult learners were trained on 90 novel L2 words and their L1 translations over 4 consecutive days (Fig. 1*a*). L2 vocabulary (concrete and abstract nouns, Supplementary Table 1) was learned in two conditions. In a gesture-performance-enriched learning condition, individuals viewed and performed gestures, while L2 words were presented auditorily. In a picture-viewing-enriched learning condition, individuals viewed pictures, while L2 words were presented auditorily (Fig. 1*b*). Gestures and pictures were congruent with word meanings. We selected the gesture performance enrichment and picture viewing enrichment conditions for two main reasons. First, of four enrichment conditions previously tested in adults (Mayer et al. 2015), only these two conditions benefitted posttraining L2 translation compared with auditory-only learning. Second, benefits of gesture performance enrichment and picture viewing enrichment were associated with responses in two different visual cortical areas, that is, the bmSTS for gesture performance enrichment and the LOC for picture viewing enrichment (Mayer et al. 2015). For succinctness, we hereafter refer to the gesture performance enrichment condition as the “gesture enrichment” learning condition, and the picture viewing enrichment condition as the “picture enrichment” learning condition. We used TMS to target the bmSTS, as the bmSTS was more easily accessible for TMS than the relevant cluster in the LOC, which was located ventrally and in a relatively medial part of the fusiform gyrus, that is, *x, y, z* = 33, −43, −14, Montreal Neurological Institute (MNI) space (Siebner and Ziemann 2007; Mayer et al. 2015). Depth of target structures is known to play a role in TMS effectiveness and focality (Wagner et al. 2009). TMS was applied to the bilateral bmSTS while participants translated auditorily presented L2 words into L1 at two time points: 5 days and 5 months following the start of L2 training. Participants did not perform gestures or view pictures during the TMS task. A within-participants control condition was included in each TMS session by applying both effective and sham TMS to the bilateral bmSTS (Fig. 1*c*).

Classification accuracy of neural responses within the bmSTS previously correlated with performance in a multiple-choice translation task (Mayer et al. 2015). In this task, participants selected the correct L1 translation of an auditorily presented L2 word from a list of options presented on a screen. This task was also used for the present TMS design and we refer to it as the “multiple-choice task” (Fig. 1*d*). In addition, we included an exploratory recall task in the present TMS design in which participants pressed a button as soon as the L1 translation came to their mind when hearing each L2 word (Fig. 1*d*).

We tested three main hypotheses. First, according to the predictive coding theory of multisensory learning (von Kriegstein 2012), the application of inhibitory stimulation to the bmSTS should slow down the translation of an auditorily presented L2 word in comparison with sham stimulation if the word has been learned with biological motion, as was the case in the gesture-enrichment condition. There should be no such effect if the word has been learned with pictures. Thus, we expected an interaction between learning condition (gesture and picture) and stimulation condition (effective and sham). The interaction was expected to be driven by a simple main effect of stimulation condition on the translation of gesture-enriched words. In contrast, reactivation learning theories (Nyberg et al. 2000; Wheeler et al. 2000; Fuster 2009), which assume that reactivated areas do not play a functional role in recognition, would predict no differential effects of bmSTS stimulation in contrast to sham bmSTS stimulation on the translation of auditorily presented L2 words (i.e., there would be no interaction between learning and TMS conditions).

Our second hypothesis was that bmSTS integrity would support the auditory translation of gesture-performance-enriched words at the later time point (5 months post learning) even more than the earlier time point (5 days postlearning). This hypothesis was based on the finding that gesture-performance enrichment is particularly powerful for learning outcome on time-scales of several months post learning in comparison with picture-viewing enrichment (Mayer et al. 2015), and less rapid decay of procedural compared with declarative memories (Mitchell et al. 1990; Tunney 2003). In our design, this hypothesis could be tested by examining the interaction between learning condition, stimulation type, and time point variables: We expected greater effects of bmSTS stimulation compared with sham stimulation on translation response times for gesture-enriched words at the later time point than at the earlier time point, and no effects of bmSTS stimulation compared with sham stimulation for picture-enriched words at either time point.

Our third hypothesis was that bmSTS stimulation would yield similar effects on the translation of both concrete and abstract words. This prediction was based on previous results showing that sensorimotor enrichment can benefit the learning of both word types (Macedonia 2014; Mayer et al. 2015), as well as embodied theories of semantic processing positing that cortical sensory integrity contributes to the processing of both concrete and abstract concepts (e.g., Lambon Ralph et al. 2017).

Response time was used as the dependent variable for testing our three hypotheses, because response time is the standard measure for TMS tasks: TMS typically influences response times rather than accuracy (Pascual-Leone et al. 1996; Ashbridge et al. 1997; Sack et al. 2007; Hartwigsen et al. 2017).

In addition to testing our three main hypotheses, the design allowed us to test the reliability of the previously reported finding that benefits of gesture performance enrichment exceed those of picture viewing enrichment over the long-term (Mayer et al. 2015). To this end, we examined accuracy outcomes in the multiple-choice task and predicted that overall accuracy would be greater for gesture-enriched words compared with picture-enriched words 5 months post learning.

### Participants

Twenty-two right-handed native German speakers (15 females; *M* age = 26.6 years, SD = 4.7 years, range 18–35) completed the study. The sample size was based on two previous experiments (*n* = 22 per experiment) that estimated beneficial effects of gesture and picture enrichment on L2 learning outcomes (Mayer et al. 2015, Experiments 1 and 2).

None of the participants reported a history of neurological, psychiatric, or psychological disorders, head injury, or any contraindications for TMS or magnetic resonance imaging (MRI). All participants reported normal hearing and normal or corrected-to-normal vision. None of the participants were raised in bilingual households. Of 32 total participants who registered for the study, one participant experienced an adverse reaction to TMS (convulsive syncope) and did not complete the testing. Syncope is the most common adverse reaction to TMS; the exact prevalence is unknown (Rossi et al. 2009). Another participant completed the training sessions but did not start the initial TMS session for medical reasons. Four participants were excluded because they were unable to return for additional testing sessions that occurred 5 months following the initial testing sessions due to time constraints, and 4 other participants were excluded due to poor localization of right or left bmSTS coordinates in individual participant space.

Written informed consent was obtained from all participants prior to the study. Participants were informed that the goal of the study was to test the effectiveness of different vocabulary learning strategies in adulthood, but they were naïve to the specific hypotheses. All participants were evaluated by a medical doctor prior to the study in order to be approved for TMS and MRI. The study was approved by the ethics committee of the University of Leipzig.

### Stimuli

Stimuli consisted of 90 pseudowords (Supplementary Table 1). The pseudowords were derived from an artificial L2 corpus referred to as “Vimmi”, developed by Macedonia and colleagues (Macedonia and Knösche 2011) and intended for use in experiments on L2 learning. The corpus was created in order to control for participants’ prior knowledge of L2s and for differences between words (e.g., length, frequency) in natural languages. Vimmi words conform to rules of Italian phonotactics (words sound like Italian but do not exist in the Italian language). All Vimmi words used in the current study were composed of three syllables consisting of vowels and consonants.

The 90 Vimmi words and 90 German translations used in the current study were previously evaluated by Mayer et al. (2015). Half of the 90 words were concrete nouns and the other half were abstract nouns. The concrete nouns referred to objects that can be perceived visually (Paivio et al. 1968; Mayer et al. 2017). Concreteness ratings (on a 0 to 10 scale) derived from a corpus of German lemmas (Köper and Schulte im Walde 2016) were significantly higher for the 45 concrete words (*M* = 6.7, SD = 0.9) than for the 45 abstract words (*M* = 3.1, SD = 0.7), *t*(88) = 22.22, *P* < 0.001, ${\eta}^2=0$.82. Imageability ratings (on a 0 to 10 scale) derived from the same corpus were also significantly higher for the 45 concrete words (*M* = 6.6, SD = 0.9) than for the 45 abstract words (*M* = 3.5, SD = 0.9), *t*(88) = 16.66, *P* < 0.001, ${\eta}^2=0$.76. Imageability refers to the ease with which a word gives rise to a sensorimotor mental image (Paivio 1971). Concreteness and imageability ratings are shown in Supplementary Table 2. Lengths of concrete and abstract German words did not significantly differ (concrete *M* = 2.4 syllables, SD = 0.8 syllables; abstract: *M* = 2.7 syllables, SD = 0.9 syllables), *t*(88) = 1.63, *P* = 0.11. Frequency of the concrete and abstract words in written German also did not significantly differ (concrete frequency score: *M* = 11.0, SD = 1.2, range 9 to 13; abstract frequency score: *M* = 11.0, SD = 1.0, range: 9 to 13), *t*(88) = 0.17, *P* = 0.87 (http://wortschatz.uni-leipzig.de/de).

#### Videos, Pictures, and Audio Files

For each of the 90 Vimmi words, a 4-s color video was created using a Canon Legria HF S10 camcorder (Canon Inc.). In each video, an actress performed a gesture that conveyed a word meaning. The actress was always positioned in the center of the video recording. She performed the gestures using head movements, movements of one or both arms or legs, fingers, or combinations of these body parts and maintained a neutral facial expression throughout each video. The word “bottle”, for example, was represented by the actress miming drinking from an imaginary bottle, and the word “good deed” was represented by the actress miming laying a donation in the imaginary hat of a homeless individual. The actress began and ended each gesture by standing motionless with her arms at her sides. Large gestures (e.g., steps or jumps) were restricted to a 1 m radius around the body’s starting position. Gestures used to convey the meanings of abstract words were agreed upon by three independent raters (Mayer et al. 2015).

To quantify differences in the iconicity of gestures associated with concrete and abstract words, 24 native German speaking participants (14 females, *M* age = 26.6 years, SD = 2.6 years, range 23–33) who did not participate in the main study rated each gesture in terms of its iconicity, that is, the relationship between each gestural form and its referent (reviewed in Perniss et al. 2010). The participants were asked: “The meaning of this gesture is intended to be [L1 word]. Do you think the gesture fits this meaning?” They rated the fit of the gesture on a 7-point Likert scale (1 = no fit, 7 = high fit; Ortega et al. 2017). The ratings were performed in an offline questionnaire and videos were provided via a file-sharing site. The fit of the concrete word gestures was rated as significantly higher (*M* = 5.2, SD = 0.6) than the fit of the abstract word gestures (*M* = 4.4, SD = 0.6), *F*_1,23_ = 122.96, *P* < 0.001, ${\eta}^2=$0.84. Iconicity ratings of the 90 gestures are shown in Supplementary Table 2.

A black-and-white line drawing was created by a professional illustrator (https://www.klaus-pitter.com/) for each of the 90 Vimmi words. Pictures conveyed word meanings by portraying humans, objects, or scenes. Pictures illustrating concrete nouns were mostly drawings of single objects, and pictures illustrating abstract nouns were often scenes. The complexity of the illustrations for concrete and abstract words was not matched since similar differences are expected in natural teaching settings.

The same actress that carried out the gestures in the videos spoke the Vimmi and the German words in audio recordings. Words were recorded using a Rode NT55 microphone (Rode Microphones) in a sound-damped chamber. The actress is an Italian native speaker and recorded the Vimmi words with an Italian accent to highlight the L2 aspect of the stimuli for German-speaking participants. Vimmi audio stimuli ranged from 654 to 850 ms in length (*M* = 819.7 ms, SD = 47.3 ms). For more details on the video, picture and audio files used in the current study, see Mayer et al. (2015). Sample stimuli can be found at http://kriegstein.cbs.mpg.de/mayer_etal_stimuli/.

### Experimental Design

The study utilized a 2 × 2 × 2 × 2 repeated-measures design. Within-participant independent factors were learning enrichment condition (gesture and picture), TMS condition (effective stimulation, sham stimulation), testing time point (5 days, 5 months), and L2 vocabulary type (concrete, abstract). Participants received both effective and sham TMS within the same session at each time point. The order in which effective and sham TMS conditions were administered within each session was counterbalanced across participants within each time point and between time points.

### Procedure

#### L2 Vocabulary Learning

Participants learned L2 words in two conditions. In the gesture learning condition, individuals viewed and performed gestures while L2 words were presented auditorily. In the picture learning condition, individuals viewed pictures while L2 words were presented auditorily. Each day of learning comprised four 33-min learning blocks. Blocks alternated between gesture and picture enrichment conditions. Each of the 45 Vimmi words included in a single learning block was repeated 4 times per block, yielding a total of 180 randomly ordered trials per block. Participants took breaks of 10 min between blocks. During breaks, participants conversed with each other and consumed snacks and drinks that were provided. Enrichment condition orders were counterbalanced across participants and learning days.

Participants were instructed prior to the start of learning that the goal was to learn as many Vimmi words as possible over the 4 days of training. Participants received no further instruction during the training except to be informed about which learning condition would occur next (i.e., gesture or picture enrichment). Since the L2 vocabulary learning took place in groups of up to 4 individuals, training sessions occurred in a seminar room with a projector and a sound system. Audio recordings were played via speakers located on each side of the screen. The volume of the playback was adjusted so that all participants could comfortably hear the words.

The assignment of the 90 stimuli to learning enrichment conditions was counterbalanced such that half of the participants learned one set of 45 words (22 concrete words and 23 abstract words) in the gesture learning condition and the other set of 45 words (23 concrete words and 22 abstract words) in the picture learning condition. The other half of the participants received the reverse assignment of the same stimuli to gesture and picture conditions. This manipulation ensured that each Vimmi word was equally represented in both the gesture enrichment condition and the picture enrichment condition, and that each concrete and abstract word was equally represented in both enrichment conditions across participants.

In each gesture enrichment trial (Fig. 1*b*), participants first heard an L2 word accompanied by a video of an actress performing a gesture that conveyed the meaning of the word (shown for 4 s). They then heard the native language (L1) translation paired with a blank screen. Finally, the L2 word was presented a second time, again accompanied by the same video of the actress performing the gesture. Participants were asked to enact the gesture along with the actress during the second showing of each video. They were free to perform the gestures mirror-inverted or they could use their right arm when the actress in the video used her right arm, for example; they were asked to use only one of the two strategies throughout the learning period. In each picture enrichment trial (Fig. 1*b*), participants first heard an L2 word accompanied by a picture that conveyed the meaning of the word (shown for 4 s). They then heard the L1 translation paired with a blank screen. Finally, the same L2 word was presented a second time, again accompanied by the same picture. A motor task was not included in the picture enrichment condition as the enrichment of picture viewing with motor information (e.g., tracing an outline of presented pictures) has been shown to be less beneficial for learning than simply viewing the pictures without performing a motor task (Mayer et al. 2015). We therefore did not combine picture enrichment with motor performance in the current study. Participants stood during all learning blocks. Standing locations during the training were counterbalanced over the 4 learning days. No information other than the L2 words, their L1 translations, and the gestures or pictures was provided to the participants for learning the L2 words. The L1 translation was always present during learning, reducing potential ambiguity of the pictures and gestures paired with the abstract words.

On days 2, 3, and 4 of the L2 vocabulary learning, participants completed paper-and-pencil vocabulary tests prior to the training, shown in Fig. 1*a*. We included these tests in order to maintain the same L2 training procedure used by Mayer et al. (2015). Participants completed free recall, L1 translation, and L2 translation tests on each of the three days. The participant with the highest combined scores on the paper-and-pencil vocabulary tests across days 2, 3, and 4 was rewarded with an additional 21€ beyond the total study compensation of 211€. Participants were informed about the financial incentive on day 1 prior to the start of the learning blocks.

Prior to vocabulary learning on day 1, participants completed three psychological tests examining their concentration ability (Concentration test; Brickenkamp 2002) (*M* score = 211.6, SD = 51.1), speech repetition ability (Nonword Repetition test; Korkman et al. 1998) (*M* score = 98.2, SD = 8.8), and verbal working memory (Digit Span test; Neubauer and Horn 2006) (*M* score = 18.7, SD = 3.7). None of the participants were outliers (2 SD above or below the group mean) with respect to their scores on any of the three tests, and all participants performed within the norms of the Concentration test for which norms were available. Participants also completed a questionnaire on their prior knowledge of L2ss and language learning experience.

#### TMS Translation Tasks

Participants performed the recall and the multiple-choice task (Fig. 1*d*) while undergoing effective and sham TMS in two TMS sessions (5 days and 5 months following the start of L2 vocabulary learning). The two tasks were performed in four 6-min blocks, each containing 45 words that had been presented on days 1 to 4. Each of the 90 words learned during the learning days was presented twice per TMS session, for a total of 180 test trials per TMS session and task. Effective and sham stimulation alternated across blocks, with half of the participants receiving effective stimulation during the first block and the other half receiving sham stimulation during the first block. Stimuli were ordered randomly within effective and sham stimulation blocks.

Each trial began with the written instruction “Press the button as soon as you know the translation” presented for 1.5 s on a screen. This was followed by the auditorily presented L2 word accompanied by a black screen. A train of seven TMS pulses at 10 Hz delivered to the bilateral bmSTS began 50 ms after the onset of each word. Participants responded as soon as they recalled the L1 translation of the L2 word by pressing a button with their right index finger (recall task, Fig. 1*d*). If they did not know the L1 translation, they did not respond. Three seconds following L2 word onsets, a screen with four response options appeared and participants were given up to 2 s to select the correct L1 translation (multiple-choice task, Fig. 1*d*). The fourth response option was always “Unknown/Other word”; participants were told to select this option if they did not know the L1 translation or thought that the correct translation was different from the three options presented. They responded by pressing one of four buttons on the response pad with their index, middle, ring, or little fingers. Even if participants did not know the translation of the L2 word after hearing it, they were still able to select one of the four options presented. Responses were considered correct if participants pressed the correct button while the response screen was present. Participants were instructed to always respond as quickly and as accurately as possible. Each trial ended with a jittered interstimulus interval (0.5–1 s) paired with a black screen. Following the first TMS session, participants completed a questionnaire on strategies that they used to learn and remember the L2 words (see “Questionnaire Results” in the Supplementary Material).

Several months following the first TMS session, participants were invited to participate in a second TMS session. The second session occurred approximately 18 weeks (*M* = 18.0 weeks, SD = 1.4 weeks) following the first session. Participants completed the same two tasks as during the first TMS session while again undergoing effective and sham stimulation. Following the second TMS session, participants completed a questionnaire on strategies they used to remember meanings of the L2 words during the second session (see “Questionnaire Results” in the Supplementary Material).

Finally, participants returned to complete the pencil-and-paper vocabulary tests (free recall, L1 translation, and L2 translation) 2–6 days (*M* = 4.1 days, SD = 1.3 days) after their second TMS session. Participants had no knowledge of the additional TMS and behavioral sessions until they were contacted a few weeks prior to their 5-month target testing dates. This was done to avoid potential rehearsal of the vocabulary during the 5-month interval between testing time points.

### Transcranial Magnetic Stimulation

#### Neuronavigation

Stereotactic neuronavigated TMS was performed using Localite software (Localite GmbH). Neuronavigation based on structural neuroimaging data from individual participants allows precise positioning of TMS coils. T1-weighted MRI scans for each participant were obtained with a 3-Tesla MAGNETOM Prisma-fit (Siemens Healthcare) using a magnetization-prepared rapid gradient echo sequence in a sagittal orientation (repetition time = 2300 ms, echo time = 2.98 ms, inversion time = 900 ms, flip angle = 9°, voxel size = 1 × 1 × 1 mm).

Structural T1 brain scans used for TMS neuronavigation were obtained from all participants prior to the TMS sessions. During each TMS session, participants were coregistered to their T1 scans. The two stimulation coils used in the current study were placed over Localite-indicated entry points of the respective target sites on the scalp. Entry points were those coordinates on each participant’s scalp that were the shortest distance to the target neural coordinates (right and left bmSTS). To stimulate the bmSTS bilaterally, a tangential coil orientation of 135° to the sagittal plane was applied with current flow within both stimulation coils reversed, resulting in a posterior to anterior (PA) current flow in the brain. A 135° coil orientation with a PA current flow is equivalent to a 45° coil orientation with an anterior to posterior (AP) current flow. Coils were secured in position using fixation arms (Manfrotto 244).

Mean MNI coordinates for bilateral bmSTS stimulation were derived from the functional MRI findings of Mayer et al. (2015): right bmSTS, *x, y, z* = 55, −41, 4; left bmSTS, *x, y, z* = −54, −41, −5. Mayer et al. (2015) found that participants translated auditorily presented L2 words learned previously with gesture enrichment more accurately than L2 words learned without enrichment (auditory-only learning), referred to as a gesture enrichment benefit. Using multivariate pattern analysis, they found that a classifier trained to discriminate BOLD responses to gesture-enriched and auditory-only words showed significant classification accuracy in the bmSTS. Classifier accuracy in the bmSTS positively correlated with the gesture enrichment benefit, suggesting a role of this area in improving learning outcomes following multisensory learning. In the current study, we stimulated the mean location across participants that demonstrated maximal classifier accuracy within the bmSTS. To ensure precise individual stimulation of target coordinates, mean MNI coordinates for the two target sites (right and left bmSTS) were transferred into individual subject space using SPM8 (Wellcome Trust Center for Neuroimaging, University College London, http://www.fil.ion.ucl.ac.uk/spm/).

#### TMS Parameters

Two MagPro X100 stimulators (MagVenture A/S) and a total of four focal figure-of-eight coils (C-B60; outer diameter = 7.5 cm) were used for stimulation. Signal software version 1.59 (Cambridge Electronic Design Limited) was used to control the TMS pulse sequence. Presentation software (Neurobehavioral Systems Inc.) was used for stimulus delivery, response recording, and to trigger TMS pulses.

An EIZO 19” LCD monitor approximately 1 m in front of the seated participant displayed task-related text (white letters, font: Arial, font size: 32 pt; black background). Shure SE215 sound isolating in-ear headphones (Shure Europe) were used to deliver L2 word recordings during the TMS sessions. Sound volume was individually adjusted prior to beginning the TMS task.

During each TMS session, a within-participants control condition was included by applying not only effective TMS to the bilateral bmSTS but also sham TMS. Sham TMS coils for each hemisphere were positioned at a 90° angle over each stimulation coil, as shown in Fig. 1*c*, and therefore did not effectively stimulate the brain. The two effective and two sham TMS coils remained in position above the scalp during all experimental blocks. Thus, the positioning of both effective and sham coils and corresponding tactile information was identical during both effective and sham stimulation, which alternated block by block. Coil locations were monitored and adjusted for head movements during the TMS sessions. The repetitive TMS protocol used (a seven-pulse train of 10 Hz TMS) was in line with published TMS safety guidelines (Rossi et al. 2009).

Prior to the TMS translation task, each participant’s individual stimulation intensity was determined by measuring their resting motor threshold (RMT). To measure RMT, we stimulated the hand region of the left primary motor cortex (M1) using single-pulse TMS, resulting in the conduction of motor-evoked potentials (MEPs) in the relaxed first dorsal interosseous muscle (FDI) of the right hand. The RMT was defined as the lowest stimulation intensity producing 5 MEPs out of 10 consecutive TMS pulses that exceeded a 50 mV peak-to-trough amplitude. A meta-analysis by Mayka et al. (2006) provided mean stereotactic coordinates of the left M1 (*x, y, z* = −37, −21, 58 mm, MNI space), which were used as a starting point to locate the M1 FDI hotspot. The coil used to elicit MEPs was oriented at 45° to the sagittal plane, inducing a PA current flow in the brain.

Effective and sham TMS intensity during the L2 translation task was set to 90% of each participant’s RMT. The same intensity was used for both TMS sessions for each participant (*M* = 40.1% of maximum stimulator output, SD = 5.6%).

### Data Analysis

All participants who completed the study (*n* = 22) were included in the analyses.

#### Analysis of Response Times in the Translation Tasks

Participants indicated that they recalled the L1 translation prior to the appearance of the four response options during fewer than half of all trials across the two TMS sessions (*M* = 41.7% of trials, SE = 4.5%), leaving an insufficient number of trials for analysis of the recall task. An exploratory analysis of these data can be found in the Supplementary Results (“Analysis of response times in the exploratory recall task”). In contrast, in the multiple-choice task, participants selected a translation from the multiple-choice options presented on the screen during *M* = 88.6% (SE = 3.6%) of trials across the two TMS sessions. In the following, we focus the analyses on the response times for the multiple-choice task.

Response times in the multiple-choice task were computed as the time interval from the appearance of the multiple-choice options on the screen until the response. Trials in which participants did not respond following the appearance of the multiple-choice options, selected the incorrect translation, or selected the fourth response options (“Unknown/Other word”) were excluded from the response time analyses.

To test our first hypothesis (see “Study Overview and Hypotheses” subsection of the methods), we ran a two-way repeated measures analysis of variance (ANOVA) with the factors learning condition (gesture and picture) and stimulation type (effective and sham) on response times in the multiple-choice task. To evaluate whether the observed patterns of response times were due to speed-accuracy tradeoffs, we correlated response times in the multiple-choice translation task with accuracy (percent correct) for each learning condition, stimulation condition, and time point.

To test our second hypothesis (see “Study Overview and Hypotheses” subsection of the methods), we ran a three-way repeated measures ANOVA with factors learning condition (gesture and picture), stimulation type (effective and sham), and time point (day 5 and month 5) on response times in the multiple-choice task. To test whether the learning condition and stimulation type variables interacted at each time point, we followed up this three-way ANOVA with two-way repeated measures ANOVAs with factors learning condition (gesture and picture) and stimulation type (effective and sham) performed separately on data from each time point (day 5 and month 5).

To test our third hypothesis (see “Study Overview and Hypotheses” subsection of the methods), we ran a four-way repeated measures ANOVA on response times in the multiple-choice task with factors learning condition (gesture and picture), stimulation type (effective and sham), testing time point (day 5 and month 5), and vocabulary type (concrete and abstract). We additionally evaluated the relation between gesture iconicity ratings and TMS effects on concrete and abstract words by correlating the gesture iconicity ratings for each of the L2 words with item-specific effects of bmSTS stimulation on response times (TMS—sham response time difference for each L2 word) in the multiple-choice task. Correlations were performed separately for each combination of the word type and time point factors.

Pairwise comparisons for all analyses were conducted using two-tailed Tukey’s honestly significant difference (HSD) post hoc tests.

#### Linear Mixed Effects Modeling of Response Times in the Multiple-choice Translation Task

To evaluate the robustness of the observed effects using an alternate analysis technique, we also tested our three hypotheses using a linear mixed effects modeling approach. We performed backwards model selection to select the model’s random effects structure, beginning with a random intercept by subject, a random intercept by auditory stimulus, a random slope by subject for each of the four independent factors (stimulation type, learning condition, time point, and vocabulary type), and a random slope by stimulus for the stimulation type and time point factors. We removed random effects terms that accounted for the least variance one by one until the fitted mixed model was no longer singular,that is, until variances of one or more linear combinations of random effects were no longer (close to) zero. The final model included three random effects terms: a random intercept by subject, a random intercept by stimulus, and a random slope by subject for the time point factor. Please see the Supplementary Material (“Analysis of TMS Effects Using Linear Mixed Effects Modeling” and Supplementary Table 3) for details.

#### Analysis of Response Accuracy

Besides testing our main hypotheses, the data also allowed us to test the reliability of the previous finding that benefits of gesture performance enrichment on L2 translation exceeded those of picture viewing enrichment over the long-term (Mayer et al. 2015). We ran a four-way repeated measures ANOVA on accuracy in the multiple-choice task, with the factors learning condition (gesture and picture), stimulation type (effective and sham), testing time point (day 5 and month 5), and vocabulary type (concrete and abstract), and examined all interactions involving the learning and time point factors.

In the results section, graphs depicting response times are shown in yellow and dark blue, and graphs depicting task accuracy shown in dark orange and purple.

#### Analysis of Paper-and-Pencil Vocabulary Test Performance

Participants’ scores on translation and free recall paper-and-pencil vocabulary tests were evaluated in three-way ANOVAs with the factors learning condition (gesture and picture), testing time point (day 2, day 3, day 4, month 5), and vocabulary type (concrete and abstract). To evaluate long-term changes in paper-and-pencil test performance, we computed the difference in performance between day 4 and month 5, and ran two-way ANOVAs on the paper-and-pencil translation and free recall test difference scores with the factors learning condition (gesture and picture) and vocabulary type (concrete and abstract). Further details and analysis results can be found in the Supplementary Material (“Analysis of paper-and-pencil test data”).

## Results

### Stimulation of the bmSTS Slows the Translation of Gesture-Enriched Foreign Vocabulary

**Figure 2:**
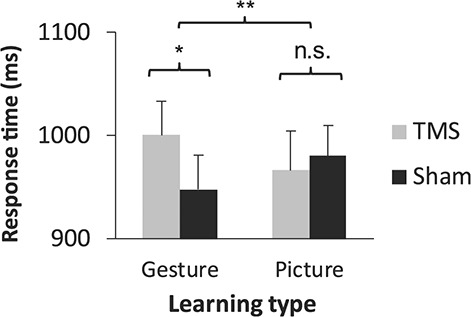
Effects of bmSTS stimulation on speed of L2 translation. Bilateral bmSTS stimulation slowed the translation of L2 vocabulary learned using gestures compared with sham stimulation in the multiple-choice task. There was no such effect for L2 vocabulary learned using pictures. The mean of each condition across time points (5 days and 5 months following the start of learning) is shown (*n* = 22 participants). Error bars represent one standard error of the mean. ^*^P < 0.05, ^*^^*^P < 0.01.

Our first and primary hypothesis was that a brain region specialized in the perception of biological motion, the bmSTS (Grossman et al. 2000), causally contributes to L2 translation following gesture-enriched L2 learning but not picture-enriched L2 learning. We therefore first tested whether bmSTS stimulation modulated L2 translation, irrespective of testing time point. The results confirmed our hypothesis. A two-way ANOVA on response times in the multiple-choice task revealed a stimulation type × learning condition interaction (*F*_1,21_ = 11.82, *P* = 0.002, two-tailed, ${\eta}_p^2= $0.36; see Supplementary Table 4 for the full set of ANOVA results). Tukey’s HSD post hoc tests revealed that response times for words that had been learned with gesture enrichment were significantly delayed when TMS was applied to the bmSTS compared with sham stimulation (*P* = 0.005, Hedge’s *g* = 0.33). This was not the case for words learned with picture enrichment. This effect was replicated in a linear mixed effects modeling analysis, described in the Supplementary Material (“Analysis of TMS Effects Using Linear Mixed Effects Modeling;” see Supplementary Table 3 for the full model results). Thus, perturbation of a brain area related to biological motion slowed the translation of L2 words that had been learned with gestures but not of L2 words learned with pictures (Fig. 2).

In a control analysis, we tested whether differences in response times under effective stimulation compared with sham stimulation conditions could be due to tradeoffs between translation speed and accuracy. Response times for correct answers in the multiple-choice translation task were correlated with accuracy (percent correct) for each learning condition, stimulation condition, and time point. If there were a speed-accuracy tradeoff, one would expect a positive correlation between response times and accuracy (i.e., the longer the response time, the greater the accuracy). Response times, however, did not correlate or correlated negatively with translation accuracy (Table 1). Thus, participants did not trade speed for accuracy.

**Table 1:** Speed-accuracy relationships in L2 translation

	Day 5		Month 5	
	TMS *r* (*P*)	Sham *r* (*P*)	TMS *r* (*P*)	Sham *r* (*P*)
Gesture	−0.84 (<0.001)^*^	−0.89 (<0.001)^*^	−0.63 (0.002)^*^	−0.34 (0.12)
Picture	−0.89 (<0.001)^*^	−0.95 (<0.001)^*^	−0.48 (0.02)	−0.46 (0.03)

Notes: In most tests, slower response times in the multiple-choice task correlated with lower translation accuracy, indicating that there was no speed-accuracy tradeoff. df = 20 for all correlations. ^*^P < 0.05, Bonferroni-corrected.

### bmSTS Supports Auditory Foreign Vocabulary Translation 5 months Post Learning

**Figure 3:**
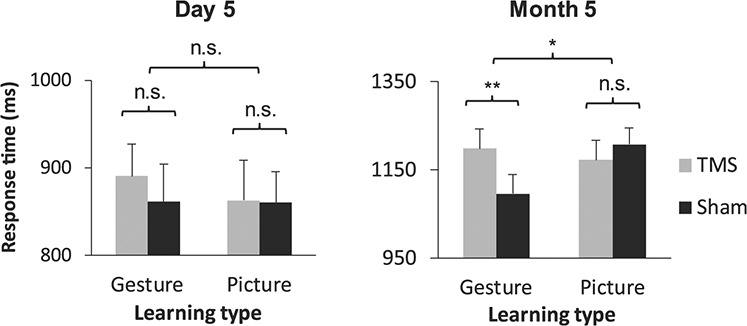
Effects of bmSTS stimulation on speed of L2 translation by time point. Effects of bmSTS stimulation on response times in the multiple-choice task occurred 5 months following learning (*n* = 22 participants). Error bars represent one standard error of the mean. ^*^P < 0.05, ^*^^*^P < 0.01.

Our second hypothesis was that bmSTS integrity would support the auditory translation of gesture-enriched words at the later time point (5 months post learning) even more than the earlier time point (5 days following the start of learning). In agreement with this hypothesis, a three-way ANOVA on response times for the multiple-choice task yielded a significant three-way stimulation type × learning condition × time point interaction (*F*_1,21_ = 7.51, *P* = 0.012, two-tailed, ${\eta}_p^2= $0.26; see Supplementary Table 5 for the full set of ANOVA results). Tukey’s HSD post hoc tests revealed a response benefit (faster responses) for words learned with gesture enrichment compared with words learned with picture enrichment under sham stimulation 5 months following learning (*P* < 0.001, Hedge’s *g* = 0.69). The application of TMS to the bmSTS negated this benefit: Response times for gesture- and picture-enriched words did not significantly differ at month 5 under effective stimulation, and responses were significantly slower under effective stimulation compared with sham stimulation for words learned with gesture enrichment (*P* = 0.001, Hedge’s *g* = 0.61). This effect was replicated in a linear mixed effects modeling analysis (see Supplementary Results, “Analysis of TMS Effects Using Linear Mixed Effects Modeling,” for details and Supplementary Table 3 for the full model results). In sum, significant effects of bmSTS stimulation on translation were more prominent 5 months following the L2 training period compared with 5 days following the start of learning (Fig. 3).

Two-way ANOVAs performed separately on data from each time point (day 5 and month 5) with the factors learning condition and stimulation type confirmed the significant learning condition × stimulation type interaction at month 5 (*F*_1,21_ = 21.81, *P* < 0.001, two-tailed, ${\eta}_p^2= $0.51; full ANOVA results shown in Supplementary Table 6) but not at day 5 (*F*_1,21_ = 1.00, *P* = 0.33, two-tailed; full ANOVA results shown in Supplementary Table 7).

### Role of Foreign Vocabulary Concreteness

**Figure 4:**
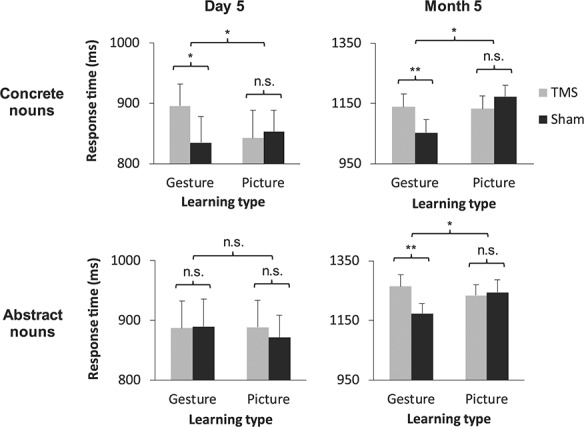
Effects of bmSTS stimulation on speed of L2 translation by time point and vocabulary type. L2 vocabulary translation response times in the multiple-choice task at the day 5 TMS session (left) and month 5 TMS session (right) by stimulation type, learning type, and vocabulary type (*n* = 22 participants). Compared with sham stimulation, stimulation of the bmSTS delayed response selection for concrete gesture-enriched nouns at day 5 and for both concrete and abstract gesture-enriched nouns at month 5. Error bars represent one standard error of the mean. ^*^P < 0.05, ^*^^*^P < 0.01.

Next, we tested our third hypothesis that the disruptive effects of bmSTS stimulation would occur independently of whether a word was classified as concrete or abstract. A four-way ANOVA on translation response times in the multiple-choice task yielded a significant four-way learning condition × stimulation type × time point × vocabulary type interaction (*F*_1,21_ = 5.24, *P* = 0.033, two-tailed, ${\eta}_p^2= $0.20; see Supplementary Table 8 for the full set of ANOVA results). Tukey’s HSD post hoc tests revealed that concrete nouns paired with gestures during learning were translated significantly more slowly during bmSTS stimulation compared with sham stimulation at day 5 (*P* = 0.005, Hedge’s *g* = 0.31; Fig. 4). Contrary to our hypothesis, this comparison was not significant for abstract nouns at day 5. At month 5, however, TMS significantly slowed the translation of both L2 word types following gesture-enriched learning (concrete words: *P* = 0.002, Hedge’s *g* = 0.44; abstract words: *P* < 0.001, Hedge’s *g* = 0.48). Response times under effective and sham stimulation did not significantly differ for words of either type that were learned in the picture enrichment condition at either time point. The unexpected four-way stimulation type × learning condition × time point × vocabulary type interaction was not significant in the mixed effects model analysis (*β* = 7.03, *t* = 1.52, *P* = 0.13, 95% CI [−2.02 16.08]; for more details, see the Supplementary Results, “Analysis of TMS Effects Using Linear Mixed Effects Modeling;” see Supplementary Table 3 for the full model results). In sum, stimulation of the bmSTS modulated the translation of the concrete gesture-enriched L2 vocabulary at the earlier time point, and the translation of both concrete and abstract gesture-enriched vocabulary at the later time point.

In a post hoc control analysis, we tested whether the subset of abstract L2 words whose translations were remembered by participants at month 5 would exhibit the same pattern of TMS effects at both time points. A three-way ANOVA on translation response times in the multiple-choice task with the factors learning condition, stimulation type, and testing time point restricted to the subset of abstract L2 words yielded a significant learning condition × stimulation type interaction (*F*_1,21_ = 4.58, *P* = 0.044, two-tailed, ${\eta}_p^2= $0.18; Supplementary Fig. 3 and see Supplementary Table 9 for the full set of ANOVA results). Tukey’s HSD post hoc tests revealed slower response times for words that had been learned with gesture enrichment when TMS was applied to the bmSTS compared with sham stimulation (*P* = 0.032, Hedge’s *g* = 0.27). This did not occur for words learned with picture enrichment. There were no significant two-way interactions and the three-way interaction was not significant (all *P*s > 0.40). Thus, for the set of abstract L2 word translations that participants remembered 5 months following training, effects of bmSTS stimulation on gesture-enriched words occurred irrespective of the testing time point.

We additionally assessed whether the effects of bmSTS stimulation on gesture-enriched words were associated with continuous ratings of gesture iconicity for each word type and time point. We correlated gesture iconicity ratings for each of the L2 words with the item-specific effects of bmSTS stimulation on response times (TMS—sham response time difference) in the multiple-choice task. Correlations were performed separately for each word type and time point. None of the correlations reached significance (all *P*s > 0.71, Bonferroni corrected), suggesting that visual properties of gestures encoded by the bmSTS did not directly correspond to overall levels of gesture iconicity.

### Sensorimotor-Enriched Training Facilitates Long-term Foreign Vocabulary Translation Accuracy

**Figure 5:**
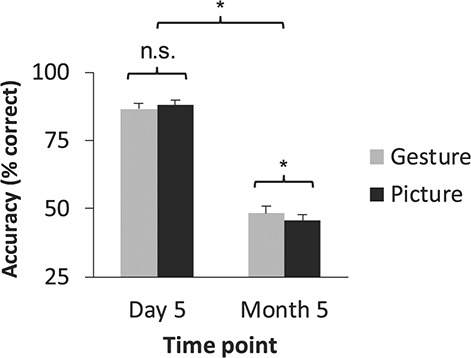
Accuracy of L2 translation following learning. Learning condition and time point variables significantly interacted in the multiple-choice task: Participants translated gesture-enriched L2 words more accurately than picture-enriched L2 words at month 5 only (*n* = 22 participants).

Besides testing our main hypotheses related to effects of TMS on translation, the data also allowed us to test the reliability of the previous finding that benefits of gesture performance enrichment on L2 translation exceed those of picture viewing enrichment over the long-term (Mayer et al. 2015). To test this, we conducted a four-way ANOVA on translation accuracy scores in the multiple-choice task (percent correct) with the factors learning condition, stimulation type, time point, and vocabulary type. The ANOVA revealed a significant learning condition × time point interaction (*F*_1,21_ = 6.86, *P* = 0.016, two-tailed, ${\eta}_p^2= $0.25; full ANOVA results shown in Supplementary Table 10). Tukey’s HSD post hoc tests revealed greater response accuracy following gesture-enriched learning compared with picture-enriched learning at month 5 (*P* = 0.035, Hedge’s *g* = 0.11), which did not occur at day 5, suggesting that gesture-enrichment-based benefits on response accuracy emerged over a period of several months (Fig. 5). This finding is consistent with the previous report that gesture enrichment outperforms picture enrichment over longer timescales (Mayer et al. 2015).

### Effects of Concreteness and bmSTS Stimulation on Foreign Vocabulary Translation Accuracy

**Figure 6:**
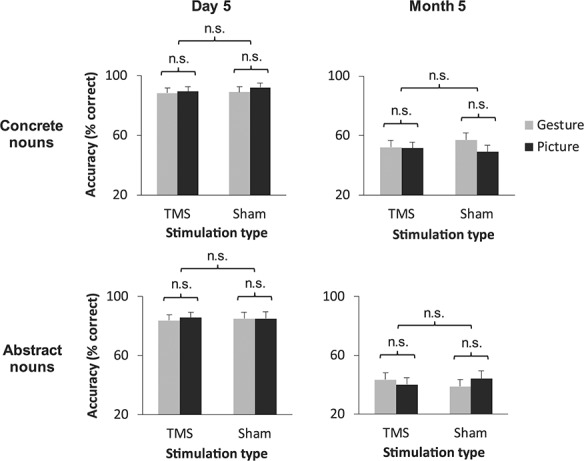
Accuracy of L2 translation in the multiple-choice task depending on learning condition, stimulation type, time point, and vocabulary type. As expected, no significant effects of TMS on L2 translation accuracy were observed at either time point (*n* = 22 participants).

For completeness, we report here further results related to multiple-choice task accuracy. There was a main effect of vocabulary type on translation accuracy (*F*_1,21_ = 35.62, *P* < 0.001, two-tailed, ${\eta}_p^2$ = 0.63) indicating that participants translated concrete words significantly more accurately than abstract words. This effect was expected based on previous studies (Macedonia and Knösche 2011; Macedonia and Klimesch 2014). Also, as expected, there were no significant effects of stimulation type on accuracy for either vocabulary type or learning condition at either time point (Fig. 6).

There was an unexpected significant four-way learning condition × stimulation type × time point × vocabulary type interaction (*F*_1,21_ = 8.23, *P* = 0.009, two-tailed, ${\eta}_p^2=$0.28) attributable to a significant difference in response accuracy between concrete and abstract gesture-enriched—but not picture-enriched—words at month 5 under sham stimulation (*P* < 0.001, Hedge’s *g* = 0.79).

## Discussion

This study revealed causal links between the integrity of specialized sensory cortices and facilitative effects of sensorimotor-enriched learning. There were three main findings. First, behavioral benefits of gesture-enriched learning were caused in part by responses within a specialized visual brain area, the bmSTS; this area was causally engaged in the auditory translation of gesture-enriched but not picture-enriched L2 words. Second, bmSTS integrity supported the auditory translation of gesture-enriched words at 5 months post learning even more than 5 days post learning. Third, bmSTS integrity supported the translation of both concrete and abstract L2 words; stimulation effects were observed for concrete nouns at the earlier time point, and for both word types at the later time point. Taken together, these findings show that sensorimotor-enriched learning constructs behaviorally relevant associations between auditory L2 words and their L1 translations by way of representations arising from specific visual cortices. Robust long-term memory representations established by sensorimotor-enriched learning can therefore be supported by task-specific, specialized sensory brain regions.

The causal relation observed between bmSTS responses and L2 translation adjudicates between influential reactivation (Nyberg et al. 2000; Wheeler et al. 2000; Fuster 2009) and predictive coding (Friston 2012; von Kriegstein 2012) theories of multisensory learning. The finding that brain responses in one sensory modality (e.g., visual) can improve task performance in another modality (e.g., auditory), depending on associations forged during learning, was expected based on predictive coding theories but not reactivation theories. Reactivation theories do not consider that reactivated regions could serve an additional purpose besides representing associatively learned stimuli (i.e., the gestures in the present study). Given that knowledge of the gestures associated with L2 words was not a requisite for accurate L2 translation in the current study, reactivation theories would posit that inhibitory stimulation of the bmSTS would not interfere with task completion. Though both gesture- and picture-enriched training involved complementary visual information, disruptive effects of bmSTS stimulation occurred only for the condition that contained stimulus information related to biological motion. Therefore, bmSTS engagement depended on sensorimotor experience. Based on predictive coding theories, we would expect motor and/or somatosensory stimulation to similarly disrupt the translation of gesture- but not picture-enriched words (for preliminary results, see Mathias et al. 2019), and expect LOC stimulation to disrupt the translation of picture- but not gesture-enriched words.

In order to recall the meaning of a newly acquired L2 word, the brain may internally simulate sensory and motor processes that were involved in learning that word. This view is consistent with the notion that the presence of additional dimensions (e.g., visual) along which stimuli can be evaluated during recognition underlies learning-by-doing-based benefits (MacLeod et al. 2010; Mathias et al. 2015). This view is also consistent with embodied theories of conceptual processing in which semantic concepts are implemented via predictive coding mechanisms (reviewed in Matheson and Barsalou 2018). Embodied accounts assume that sensory and motor brain areas are critical for representing experience-dependent features of concepts. In support of these accounts, several neurostimulation studies have revealed evidence for the functional relevance of motor areas in behavioral responses to L1 action words that refer to body movements (Pulvermüller et al. 2005; Vukovic et al. 2017). Our findings add a fundamentally novel line of research to these previous results by revealing the causal relevance of sensory brain responses in the representation of recently acquired L2 words, and, in particular, nonaction words such as concrete and abstract nouns.

Beneficial effects of gesture enrichment on L2 vocabulary learning outcomes have previously been found for a variety of word classes beyond those reported here, including verbs, adverbs, adjectives, and prepositions (Saltz and Donnenwerth-Nolan 1981; Macedonia et al. 2011; Macedonia and Knösche 2011; Macedonia and Klimesch 2014). We would expect bmSTS integrity to also causally influence the translation of gesture-enriched L2 words belonging to these other word classes. In the current study, effects of bmSTS integrity on the translation of abstract nouns appeared to differ between the two testing time points. However, when our analysis of bmSTS effects was restricted to only those abstract words whose meanings were remembered by participants 5 months following L2 training, we found that bmSTS integrity influenced gesture-enriched abstract L2 word translation across both testing time points. These results suggest that bmSTS integrity supported the translation of a subset of gesture-enriched abstract L2 words, and that effects of bmSTS stimulation on this set of words at the earlier time point were masked by responses to other abstract items. Models of L2 vocabulary learning in adults such as the semantic transfer hypothesis (Jiang 2000) propose that, initially, L1 words mediate the link between novel L2 words and their related semantic concepts, and that links between L2 wordforms and L1-linked semantic concepts gradually strengthen with training. It is likely that gestures such as moving one’s arms to throw an imaginary ball were already integrated into participant’s semantic representations for the L1 word “ball” prior to L2 training. If such gestures were already integrated into concrete L1 word representations, then these gestures could have been more efficiently linked with L2 tokens compared with the abstract word gestures, which were significantly less iconic. We speculate that learners relied on alternate strategies for translating some of the less iconic abstract L2 words, which made bmSTS contributions to the translation of concrete words at the earlier time point more readily detected than bmSTS contributions to the translation of abstract words.

Several theories of embodied semantics (e.g., the hub-and-spoke model; Lambon Ralph et al. 2017) propose that sensorimotor information is integrated into L1 conceptual representations. Whether gestural information present during enriched learning is also integrated into the representational format of L2 words, or merely provides additional information that is representationally distinct from an L2 word’s meaning, is an open question. We speculate that enriched learning may speed up the formation of links between L2 words and L1-linked semantic concepts (cf. Hald et al. 2016). In the case of abstract words, sensorimotor facilitation may function by building associations between abstract concepts and perceptible sensory and motor events within a situational context. The L2 translation of the word “innocence”, for example, is difficult to learn if paired simply with its L1 equivalent. It becomes easier to learn if paired with the gesture of shrugging one’s shoulders, even though “innocence” is not defined as shrugging (Macedonia and Knösche 2011). Abstract gesture-enriched L2 representations may therefore rely to a greater extent on imagined situational contexts than concrete gesture-enriched L2 representations, which instead may rely more on L1-linked semantic concepts (see also Barsalou 1999).

The performance of gestures during learning also yielded beneficial long-term effects on L2 translation accuracy relative to picture-enriched learning (cf. Mayer et al. 2015; Repetto et al. 2017; see also Supplementary Material, “Analysis of paper-and-pencil test data”). One possible mechanism for this long-term effect is that representations in visual cortices are part of a procedural memory system that supports knowledge of recently learned L2 words following sensorimotor-enriched learning (Macedonia and Müeller 2016). This result extends the previous finding that gesture-enriched L2 words elicit responses not only within brain regions that typically mediate declarative memory such as the hippocampus and parahippocampal gyri, but also regions associated with procedural memory such as the premotor cortex, basal ganglia, and cerebellum (Macedonia and Müeller 2016).

In our experiment, we characterize learning by doing as “sensorimotor”-enriched learning rather than “motor”-enriched learning because motor components of gesture-based enrichment can never be fully separated from associated sensory components. Even if learners were to perform self-created gestures without viewing a model, they would still receive visual feedback from their own and others’ body movements, as well as other types of movement-associated sensory feedback. The STS, in particular, appears to be sensitive to receiving visual sensory feedback that aligns with one’s self-generated movements (Limanowski et al. 2018). Learning by doing inevitably involves the integration of sensory and motor aspects of one’s experience.

Some studies have suggested that regions in the vicinity of the bmSTS are involved in the decoding of social information. For example, the STS has been shown to preferentially support viewing physical interactions between two or more agents, such as one shape pushing another shape down a hill, relative to viewing two agents perform independent actions at the same time (Castelli et al. 2000; Schultz et al. 2004; Schultz et al. 2005; Isik et al. 2017). We view it as unlikely that a social task component induced differential effects of TMS on gesture- and picture-enriched words for two main reasons. First, the kinds of social interactions previously shown to elicit responses within the STS were not a part of the training paradigm in the current study. Second, the bmSTS is also activated during the translation of L2 words that have been learned by groups of participants who simply view gestures without performing them, and no evidence of STS involvement was found following training in which groups of participants viewed pictures while performing a related motor task (Mayer et al. 2015).

Our TMS approach took advantage of the focal spatial resolution of TMS to transiently interfere with processing in a specific cortical target (Sack et al. 2007). Generally, it is argued that, if a TMS pulse affects performance, then the stimulated area provides an essential contribution to the behavior under investigation (Siebner et al. 2009). However, TMS results can also be impacted by network projection effects, in which the stimulated area projects to a critical site, or by the stimulation of nearby regions (Di Lazzaro et al. 1999; reviewed in Sandrini et al. 2011). The specificity of the TMS effects to the gesture enrichment condition in the current study suggests that regions supporting general aspects of task performance, for example, auditory processing, did not mediate those effects. There is also no indication from previous fMRI findings that auditory and visual regions in the vicinity of the bmSTS encode gesture-enriched L2 words more reliably than picture-enriched L2 words (Mayer et al. 2015). The finding that only gesture-enriched words were influenced by TMS is also not consistent with a potential placebo effect of TMS, as this would rather induce a change in performance under effective compared to sham stimulation for both gesture- and picture-enriched words. Additionally, the participants were naïve with respect to whether TMS was expected to facilitate or interfere with their performance, as well as the condition(s) on which TMS was hypothesized to have an effect. In sum, any potential differences in the perceived effectiveness of the two stimulation types were not substantial enough to systematically influence performance.

A growing literature has reported positive effects of arousal-based interventions such as physical exercise (Hötting et al. 2016), emotion regulation (Storbeck and Maswood 2016), and even music (Schellenberg et al. 2007) on cognitive task performance. Though effective, these approaches do not encode associations between different components of the word acquisition experience in the same way as gesture-enriched learning, as gestures are intrinsically bound to specific stimulus information (Markant et al. 2016). If behavioral benefits of enrichment were due solely to increased arousal during gesture-enriched learning compared to picture-enriched learning, then stimulation of a specialized visual area would not have disrupted those benefits. Further, any potential differences between gesture- and picture-enriched learning in terms of arousal were not large enough to distinguish these conditions in terms of performance accuracy immediately following learning. Previously, the combination of a motor task with picture viewing (tracing an outline of presented pictures) during L2 learning benefitted learning outcomes less than simply viewing pictures without performing any movements (Mayer et al. 2015), and the performance of semantically related gestures enhanced learning outcomes compared with the performance of meaningless gestures (Macedonia et al. 2011). These outcomes suggest that gesture enrichment benefits cannot be explained simply by the presence of movement during learning. The current results therefore steer away from more general explanations for beneficial effects of sensorimotor-enriched or multisensory-enriched learning such as increased arousal or attention. Hence, teaching strategies may be advanced by establishing links between new information and congruent sensorimotor and multisensory enrichment (cf. Andrä et al. 2020).

We conclude that beneficial behavioral effects of sensorimotor-enriched training are caused in part by responses within specialized sensory brain regions. The causal relation observed between sensory brain responses and behavioral performance significantly advances our knowledge of neuroscientific mechanisms contributing to benefits of sensorimotor-enriched learning. The results show that sensorimotor-enriched learning can be used to enhance outcomes by linking sensory brain functions with behavioral performance. The findings have consequence for the ways in which classroom teaching practices are designed, because they indicate that sensory mechanisms are a critical component of effective learning-by-doing methods.

## Funding

German Research Foundation (grant KR 3735/3-1), a Max Planck Research Group (to K.v.K.), an Erasmus Mundus Postdoctoral Fellowship (to B.M.). B.M. is also supported by the European Research Council Consolidator (grant SENSOCOM 647051 to K.v.K.).

## Notes

We thank Frieder Schillinger for comments on a previous version of the manuscript. Corresponding author address: Technical University Dresden, Chair of Cognitive and Clinical Neuroscience, Faculty of Psychology, Bamberger Str. 7, Dresden 01187, Germany. *Conflict of Interest*: None declared.

## Supplementary Material

Supplementary_File_bhaa240
