# Supplementary material for: Visual Sensory Cortices Causally Contribute to Auditory Word Recognition Following Sensorimotor-Enriched Vocabulary Training
Source: Cereb Cortex. 2020 Sep 22;31(1):513–28. doi: 10.1093/cercor/bhaa240 (PMC7727387; doi:10.1093/cercor/bhaa240)
Supplement: Supplementary_File_bhaa240 [file supplementary_file_bhaa240.pdf]

Supplementary Material

Table S1. Vocabulary used in the experiment. 90 Vimmi and German words, and their English translations. Assignment of words to the gesture learning and the picture learning conditions was counterbalanced across participants, ensuring that each Vimmi word was represented equally in both learning conditions.

Concrete nouns			Abstract nouns		
**German**	**English**	**Vimmi**	**German**	**English**	**Vimmi**
Ampel	traffic light	gelori	Absage	cancellation	munopa
Anhänger	trailer	afugi	Alternative	alternative	mofibu
Balkon	balcony	usito	Anforderung	requirement	utike
Ball	ball	miruwe	Ankunft	arrival	matilu
Bett	bed	suneri	Aufmerksamkeit	attention	fradonu
Bildschirm	monitor	zelosi	Aufwand	effort	muladi
Briefkasten	letter box	abota	Aussicht	view	gaboki
Decke	ceiling	siroba	Befehl	command	magosa
Denkmal	memorial	frinupo	Besitz	property	mesako
Eintrittskarte	entrance ticket	edafe	Bestimmung	destination	wefino
Faden	thread	kanede	Bitte	plea	pokute
Fahrrad	bicycle	sokitu	Disziplin	discipline	motila
Fenster	window	uribo	Empfehlung	recommendation	giketa
Fernbedienung	remote control	wilbano	Gedanke	thought	atesi
Flasche	bottle	aroka	Geduld	patience	dotewa
Flugzeug	airplane	wobeki	Gleichgültigkeit	indifference	frugazi
Gemälde	painting	bifalu	Information	information	sapezo
Geschenk	present	zebalo	Korrektur	correction	fapoge
Gitarre	guitar	masoti	Langeweile	boredom	elebo
Handtasche	purse	diwume	Mentalität	mentality	gasima
Kabel	cable	zutike	Methode	method	efogi
Kamera	camera	lamube	Mut	bravery	wirgonu
Kasse	till	asemo	Partnerschaft	partnership	nabita
Katalog	catalog	gebamo	Rücksicht	consideration	ukowe
Kleidung	clothes	wiboda	Sensation	sensation	boruda
Koffer	suitcase	mewima	Stil	style	lifawo
Maschine	machine	nelosi	Talent	talent	puneri
Maske	mask	epota	Tatsache	fact	botufe
Papier	paper	serawo	Teilnahme	participation	pamagu
Reifen	tire	wasute	Tendenz	tendency	pefita
Ring	ring	guriwe	Theorie	theory	sigule
Rucksack	backpack	lofisu	Therapie	therapy	giwupo
Sammlung	collection	etuko	Tradition	tradition	uladi
Schlüssel	key	abiru	Triumph	triumph	gepesa

Schublade	drawer	lutepa	Übung	exercise	fremeda
Sonnenbrille	sunglasses	woltume	Unschuld	innocence	dafipo
Spiegel	mirror	dubeki	Veränderung	change	zalefa
Straßenbahn	tram	umuda	Verständnis	sympathy	gorefu
Tageszeitung	daily newspaper	gokasu	Vorgehen	procedure	denalu
Telefon	telephone	esiwu	Vorwand	excuse	pirumo
Teller	plate	buliwa	Warnung	warning	gubame
Teppich	carpet	batewo	Wohlstand	wealth	bekoni
Verband	bandage	magedu	Wohltat	benefaction	migedu
Zelt	tent	wugezi	Zulassung	admission	frokibe
Zigarette	cigarette	zowitu	Zweck	purpose	dizela

Table S2. Concreteness and imageability ratings of the 90 words used in the experiment (derived from Köper & Schulte im Walde, 2016), and iconicity ratings of their associated gestures (*n* = 24 participants).

Concrete nouns				Abstract nouns			
**Word**	**Concrete-ness**	**Image-ability**	**Icon-icity**	**Word**	**Concrete-ness**	**Image-ability**	**Icon-icity**
airplane	7.7	7.6	6.4	admission	3.2	4.2	2.4
backpack	7.8	7.3	6.5	alternative	3.5	2.6	5.3
balcony	7.0	7.0	5.4	arrival	4.4	5.3	2.6
ball	6.8	7.1	5.0	attention	3.0	4.1	4.6
bandage	5.2	5.2	4.9	benefaction	2.1	3.5	4.0
bed	7.9	8.3	6.0	boredom	2.6	4.2	6.5
bicycle	7.6	7.7	6.4	bravery	3.0	4.4	3.5
bottle	8.0	6.7	6.2	cancellation	3.4	3.6	5.6
cable	6.5	6.6	4.5	change	2.9	2.9	4.6
camera	7.5	6.8	7.0	command	4.0	3.8	5.9
carpet	7.1	7.3	4.1	consideration	2.4	2.8	4.1
catalog	5.0	6.0	4.8	correction	4.4	3.4	4.0
ceiling	7.8	6.8	4.9	destination	2.5	2.9	4.8
cigarette	7.9	7.4	7.0	discipline	3.6	3.8	3.7
clothes	6.4	6.9	4.8	effort	2.2	2.5	3.3
collection	4.1	4.5	3.9	excuse	3.4	3.8	2.5
daily	6.3	6.9	3.5	exercise	3.7	4.1	5.8
drawer	6.1	5.5	6.5	fact	2.0	1.9	4.8
entrance ticket	5.1	5.4	5.9	indifference	2.1	3.5	6.3
guitar	6.8	6.6	6.9	information	3.6	4.1	2.3
key	6.2	6.0	6.6	innocence	3.5	4.3	6.1
letter box	6.9	6.1	6.3	mentality	1.9	2.6	3.7
machine	6.7	6.3	3.8	method	3.1	2.1	2.0
mask	6.4	6.6	4.3	participation	3.7	3.8	3.7

memorial	5.8	5.9	3.6	partnership	3.4	4.3	5.4
mirror	6.4	8.2	4.6	patience	2.1	3.5	5.5
monitor	6.7	6.5	5.3	plea	4.6	4.3	6.6
painting	6.4	6.9	4.6	procedure	3.0	2.6	3.5
paper	7.5	6.8	5.3	property	3.1	4.1	4.8
plate	8.1	8.0	5.0	purpose	2.2	1.9	2.8
present	4.9	5.9	4.4	recommendation	3.3	3.0	4.4
purse	7.7	7.1	4.8	requirement	1.8	2.3	2.6
remote control	6.2	5.7	6.1	sensation	3.5	4.4	4.2
ring	7.1	7.1	6.7	style	3.4	3.9	4.5
suitcase	7.5	7.9	5.8	sympathy	1.8	2.5	5.5
sunglasses	7.7	7.4	5.0	talent	3.4	3.7	2.0
telephone	5.8	6.2	7.0	tendency	2.7	1.9	5.1
tent	7.6	7.9	5.2	theory	3.2	3.7	2.6
thread	6.2	6.0	5.5	therapy	4.4	4.3	3.4
till	5.3	4.9	4.3	thought	2.9	3.8	6.5
tire	6.8	6.0	4.8	tradition	2.3	2.7	3.3
traffic light	6.7	6.5	2.9	triumph	2.5	5.2	5.7
trailer	5.7	5.5	3.5	view	3.7	4.9	6.3
tram	6.6	7.2	5.7	warning	3.4	3.7	4.6
window	6.7	6.8	5.8	wealth	3.0	4.8	4.9

Questionnaire results

Here we summarize participants‘ responses regarding their strategies during the TMS session tasks and the 4-day vocabulary training period.

Day 5 post-TMS questionnaire. This questionnaire was filled in after the first TMS session.

The following questions are related to the memory test that you just completed during the TMS session:

1. When I heard words that I learned with pictures, I imagined the pictures. 1 = Never, 3 = Sometimes, 5 = Very often. *M* = 3.73, *SD* = .98.

2. Imagining pictures during the test helped me to remember the German meanings of the words. 1 = Never, 3 = Sometimes, 5 = Very often. *M* = 3.27, *SD* = 1.24.

3. If you imagined any pictures during the test, how vivid were those pictures in your mind? 1 = Not at all vivid, 3 = Somewhat vivid, 5 = Extremely vivid. *M* = 2.59, *SD* = 1.58.

4. When I heard words that I learned with gestures, I imagined the videos of the gestures. 1 = Never, 3 = Sometimes, 5 = Very often. *M* = 3.59, *SD* = 1.01.

5. Imagining videos of the gestures during the test helped me to remember the German meanings of the words. 1 = Never, 3 = Sometimes, 5 = Very often. *M* = 3.18, *SD* = 1.05.

6. If you imagined videos of the gestures during the test, how vivid were those videos in your mind? 1 = Not at all vivid, 3 = Somewhat vivid, 5 = Extremely vivid. *M* = 3.41, *SD* = .85.

7. When I heard words I learned with gestures, I imagined myself performing the gestures. 1 = Never, 3 = Sometimes, 5 = Very often. *M* = 3.18, *SD* = 1.14.

8. Imaging myself performing the gesture during the test helped me to remember the German meaning of the word. 1 = Never, 3 = Sometimes, 5 = Very often. *M* = 3.41, *SD* = 1.14.

9. If you imagined yourself performing a gesture during the test, how vivid were the movements in your mind? 1 = Not at all vivid, 3 = Somewhat vivid, 5 = Extremely vivid. *M* = 3.67, *SD* = 1.02.

10. During the TMS session, did you better remember the meanings of the words you learned with pictures or the ones you learned with gestures? Pictures: 12 participants; Gestures: 10 participants.

11. The words I learned with gestures felt more familiar during the test than words I learned with pictures. 1 = Strongly disagree, 3 = Neither agree nor disagree, 5 = Strongly agree. *M* = 2.82, *SD* = .91.

12. The words I learned with pictures felt more familiar during the test than words I learned with gestures. 1 = Strongly disagree, 3 = Neither agree nor disagree, 5 = Strongly agree. *M* = 3.23, *SD* = 1.02.

The following questions are related to the training that you completed from Monday to Thursday:

13. During the training sessions, the pictures helped me to remember the German meanings of the Vimmi words. 1 = Strongly disagree, 3 = Neither agree nor disagree, 5 = Strongly agree. *M* = 4.14, *SD* = .94.

14. During the training sessions, the gestures helped me to remember the German meanings of the Vimmi words. 1 = Strongly disagree, 3 = Neither agree nor disagree, 5 = Strongly agree. *M* = 3.77, *SD* = 1.02.

15. During the training sessions, performing gestures helped me learn better than viewing pictures. 1 = Strongly disagree, 3 = Neither agree nor disagree, 5 = Strongly agree. *M* = 3.32, *SD* = 1.21.

16. During the training sessions, viewing pictures while learning helped me learn better than performing gestures. 1 = Strongly disagree, 3 = Neither agree nor disagree, 5 = Strongly agree. *M* = 2.91, *SD* = 1.23.

17. The gestures in the videos were gestures that I would think of myself if I had to "enact" the word. 1 = Strongly disagree, 3 = Neither agree nor disagree, 5 = Strongly agree. *M* = 3.50, *SD* = .91.

18. While watching the video with the actress, I had to remind myself not to think of other gestures that I could perform. 1 = Strongly disagree, 3 = Neither agree nor disagree, 5 = Strongly agree. *M* = 1.95, *SD* = 1.09.

Month 5 post-TMS questionnaire. This questionnaire was filled in after the second TMS session.

1. Have you actively rehearsed the vocabulary since the training week and the first TMS session? 1 = Never, 3 = Sometimes, 5 = Very often. *M* = 1.27, *SD* = .55.

2. Have you thought about the vocabulary learned in the study? 1 = Never, 3 = Sometimes, 5 = Very often. *M* = 2.45, *SD* = 1.01.

3. Have you actively rehearsed the gestures since the training week and the first TMS session? 1 = Never, 3 = Sometimes, 5 = Very often. *M* = 1.05, *SD* = .21.

4. Have you thought about the gestures learned in the study? 1 = Never, 3 = Sometimes, 5 = Very often. *M* = 2.23, *SD* = .87.

5. Have you thought about the videos that you saw in the study? 1 = Never, 3 = Sometimes, 5 = Very often. *M* = 2.09, *SD* = .75.

6. Have you thought about the pictures that you saw in the study? 1 = Never, 3 = Sometimes, 5 = Very often. *M* = 2.14, *SD* = 1.04.

7. Have you learned or started learning another foreign language since the training week and the first TMS session? No: 22 participants. Yes: 0 participants.

8. Can you still remember the German words that you learned? 1 = None, 3 = Some, 5 = Very many. *M* = 3.19, *SD* = 1.03.

9. Can you still remember the Vimmi words that you learned? 1 = None, 3 = Some, 5 = Very many. *M* = 2.38, *SD* = .92.

10. Can you still remember the German-Vimmi or Vimmi-German translations? 1 = None, 3 = A few, 5 = Very many. *M* = 2.24, *SD* = .89.

11. How easy or difficult is it for you to remember the vocabulary learned with gestures? 1 = Very easy, 3 = Medium, 5 = Very difficult. *M* = 3.14, *SD* = .96.

12. How easy or difficult is it for you to remember the vocabulary learned with pictures? 1 = Very easy, 3 = Medium, 5 = Very difficult. *M* = 3.14, *SD* = 1.15.

Analysis of response times in the exploratory recall task

In the recall task, response time was defined as the time elapsed between the start of the auditory L2 word presentation and the participant’s indication by button press (prior to the appearance of the four response options) that they knew the L1 translation of the presented L2 word. Participants indicated that they recalled the L1 translation prior to the appearance of the

four response options during fewer than half of all trials across the two TMS sessions (*M* = 41.7% of trials, *SE* = 4.5%), leaving an insufficient number of trials for analysis of this exploratory task component. We nevertheless explored the data and analyzed the recall response times for correct response trials. In order to evaluate recall response times for correct response trials, we analyzed trials in which participants first indicated by button press that they recalled the L1 translation and subsequently selected the correct translation from the list of response options presented on the screen.

A four-way ANOVA on recall response times for correct response trials with factors learning condition, stimulation type, time point, and vocabulary type yielded a significant main effect of time point, ($F_{1, 21} = 86.66$, $p < .001$, two-tailed, $\eta_p^2 = .80$). Recall response times were significantly faster at day 5 than month 5. There was, however, no significant main effect of vocabulary type ($p = .96$), which was one of the most robust effects throughout our other dependent measure, i.e., the multiple choice task reported in the main manuscript. Recall response times for concrete words (*M* = 1527 ms, *SE* = 26 ms) did not differ from response times for abstract words (*M* = 1526 ms, *SE* = 23 ms). The ANOVA yielded a significant learning condition × vocabulary type interaction ($F_{1, 21} = 6.52$, $p = .019$, two-tailed, $\eta_p^2 = .24$), and significant learning condition × time point × vocabulary type interaction ($F_{1, 21} = 4.38$, $p = .049$, two-tailed, $\eta_p^2 = .17$). However, Tukey's HSD post-hoc tests revealed no significant differences between concrete and abstract noun response times within any time point or learning condition. The predicted two-way interaction between learning condition and stimulation type variables was also not significant ($p = .49$): Response times did not significantly differ between any conditions (TMS-Gesture: *M* = 1506 ms, *SE* = 33 ms; Sham-Gesture: *M* = 1536 ms, *SE* = 32 ms; TMS-Picture: *M* = 1532 ms, *SE* = 37 ms; Sham-Picture: *M* = 1533 ms, *SE* = 36 ms). There were no other significant main effects or interactions.

Given that not even the robust difference between concrete and abstract vocabulary types emerged in this analysis of recall response times, we assume that the low response rate yielded an insufficient number of trials for analysis of this task component. An alternative interpretation is that there was no effect of bmSTS stimulation on this specific vocabulary task.

Analysis of TMS effects using linear mixed effects modeling

Methods. To evaluate the robustness of the observed TMS effects using an alternate analysis technique, we also tested our three hypotheses using a linear mixed effects modeling approach. Linear mixed effects models were generated in R version 1.2.1335 using the 'lme4' package (Bates, Maechler, Bolker, & Walker, 2015). To select the random effects structure, we performed backwards model selection, beginning with a random intercept by subject, a random intercept by auditory stimulus, a random slope by subject for each of the four independent factors (stimulation type, learning condition, time point, and vocabulary type), and a random slope by stimulus for the stimulation type and time point factors. We removed random effects terms that accounted for the least variance one by one until the fitted mixed model was no longer singular, i.e. until variances of one or more linear combinations of random effects were no longer (close to) zero. The final model included three random effects terms: a random intercept by subject, a random intercept by stimulus, and a random slope by subject for the time point factor.

Contrasts were coded using simple coding, i.e. ANOVA-style coding, such that the model coefficient represented the size of the contrast from a given predictor level to the (grand) mean (represented by the intercept). The dependent measure was response times in the multiple choice translation task. Significance testing was performed using Satterthwaite's method implemented in the 'lmerTest' package, with an alpha level of $\alpha = 0.05$ (Kuznetsova, Brockhoff, & Christensen, 2017). Post-hoc Tukey tests were conducted using the 'emmeans'

package (Lenth, Singmann, Love, Buerkner, & Herve, 2019). The full model results are shown in **Table S3**.

Results.

Hypothesis 1. We first examined whether bmSTS stimulation modulated L2 translation. The model revealed a significant interaction of stimulation type and learning condition factors (β = -15.43, t = -3.34, p < .001, 95% CI [-24.50 -6.37]), confirming our first hypothesis. Tukey's HSD post-hoc tests revealed that response times for words that had been learned with gesture enrichment – but not picture enrichment – were significantly delayed when TMS was applied to the bmSTS compared to sham stimulation (β = -42.99, p = .006).

Hypothesis 2. We next examined whether bmSTS integrity supported the auditory translation of gesture-enriched words at the later time point (5 months post-learning) even more than the earlier time point (5 days following the start of learning). The model revealed a significant three-way interaction of stimulation type, learning condition, and time point variables (β = -12.41, t = -2.69, p < .001, 95% CI [-21.48 -3.35]). Tukey's HSD post-hoc tests revealed a response benefit (faster responses) for gesture-enriched learning compared to picture-enriched learning under sham stimulation 5 months following learning (β = -85, p = .001). The application of TMS to bmSTS negated this benefit: Responses were significantly slower for the gesture condition at month 5 during TMS compared to sham stimulation (β = -68, p = .023).

Hypothesis 3. Finally, we examined whether the disruptive effects of bmSTS stimulation would occur independent of the conceptual perceptibility of the L2 word referents (i.e., whether a word was concrete or abstract). The four-way stimulation type × learning condition × time point × vocabulary type interaction was not reliable in the fitted model (β = 7.03, t = 1.52, p = .13, 95% CI [-2.02 16.08]), suggesting that effects of bmSTS stimulation did not significantly differ across vocabulary types.

In sum, linear mixed modeling yielded the same results as the ANOVA-based approach reported in the main manuscript, with the exception of the four-way interaction, which was significant in the ANOVA but did not reach significance in the mixed model analysis.

Table S3. Linear mixed effects regression testing the effects of stimulation type, learning condition, time point, and vocabulary type on response times in the multiple choice translation task. *$p < .05$, **$p < .01$, ***$p < .001$.

Fixed effects						*Random effects*			
	Estimate	*SE*	*t*	*p*	CI			Variance	*SD*
Intercept	1032	32.42	31.82	<.001	965.66, 1098.10	Participant	Intercept	21325	146.03
Stimulation	6.07	4.62	1.31	.19	-3.00, 15.13		Time	3674	60.61
Learning condition	3.23	4.63	.70	.49	-5.85, 12.31	Stimulus	Intercept	5061	71.14
Time point	161.5	1.39	11.65	<.001***	132.69, 189.71				
Vocabulary	-32.79	6.99	-4.69	<.001***	-46.52, -18.83				
Stimulation × Learning	-15.43	4.62	-3.34	<.001***	-24.50, -6.37				
Stimulation × Time	-.009	4.63	-.002	.99	-9.08, 9.07				
Learning × Time	11.51	4.62	2.49	.013*	2.44, 20.57				
Stimulation × Vocabulary	-.69	4.61	-.15	.88	-9.73, 8.36				
Learning × Vocabulary	7.26	4.63	1.57	.12	-1.82, 16.35				
Time × Vocabulary	-29.12	4.70	-6.20	<.001***	-38.34, -19.91				
Stimulation	-12.41	4.62	-2.69	.007**	-21.48,				

× Learning × Time					-3.35
Stimulation × Learning × Vocabulary	-4.55	4.62	-.98	.33	-13.60, 4.51
Stimulation × Time × Vocabulary	-3.38	4.62	-.73	.46	-12.42, 5.67
Learning × Time × Vocabulary	4.94	4.63	1.07	.29	-4.14, 14.01
Stimulation × Learning × Time × Vocabulary	7.03	4.62	1.52	.13	-2.02, 16.08

Analysis of paper-and-pencil test data

Methods. Participants completed paper-and-pencil vocabulary tests (free recall, L1 translation, and L2 translation tests) on days 2, 3, and 4 of the L2 vocabulary training period and at 5 months post-training. During the translation tests, participants received a list of either the 90 German words (L1 translation test) or the 90 Vimmi words (L2 translation test) and were asked to write the correct translation next to each word. During the free recall test, participants received a blank sheet of paper and were asked to write down as many German words, Vimmi words, or combination of a Vimmi word with its German translation that occurred during the learning as they could remember. The free recall test was always administered before the translation tests, and the order of the two translation tests was counterbalanced across days and participants.

Paper-and-pencil tests were independently scored for accuracy by two raters. L1 and L2 translation tests were scored in terms of the total number of correct translations recalled in each test (one point for each correct translation). A Vimmi word was considered correct if the two

independent raters agreed that the word that was written down was valid for the sound pronounced in the audio file according to German sound-letter mapping. A German word was considered correct if a participant wrote down the German word that was assigned to the Vimmi word during learning or if a participant wrote down a synonym of the German word, according to a standard German synonym database (www.duden.de). Free recall tests were scored by counting the number of correct translations (German-Vimmi or Vimmi-German word pairs), German words that were missing corresponding Vimmi words, and Vimmi words that were missing corresponding German words. Three points were given for each correct translation (German-Vimmi or Vimmi-German word pair). One point was given for each correctly-recalled German word that was missing a corresponding Vimmi translation and vice versa.

Effects of enrichment on paper-and-pencil vocabulary test accuracy.

Translation tests. To analyze the translation tests, percentages of correctly translated words were averaged across the two tests (as in Mayer et al., 2015) and submitted to a three-way ANOVA with the factors learning condition (gesture, picture), testing time point (day 2, day 3, day 4, month 5), and vocabulary type (concrete, abstract). The ANOVA did not yield any interactions of the learning condition factor with other variables, suggesting similar effects of gesture- and picture-enriched learning on vocabulary test performance. There was a significant main effect of testing time point ($F_{3, 63} = 94.28$, $p < .001$, two-tailed, $\eta_p^2 = .82$). Tukey's HSD post-hoc tests revealed that overall test scores at each time point differed significantly from test scores at all other time points (all *p*s < .001, Hedge's *g* range: 0.62 to 2.55; **Fig. S1a**). The ANOVA additionally yielded a significant main effect of vocabulary type ($F_{1, 21} = 135.17$, $p < .001$, two-tailed, $\eta_p^2 = .87$) and a significant time point × vocabulary type interaction ($F_{3, 63} = 5.78$, $p = .001$, two-tailed, $\eta_p^2 = .22$). Overall, test scores were significantly higher for concrete nouns compared to abstract nouns. There were no other significant main effects or interactions.

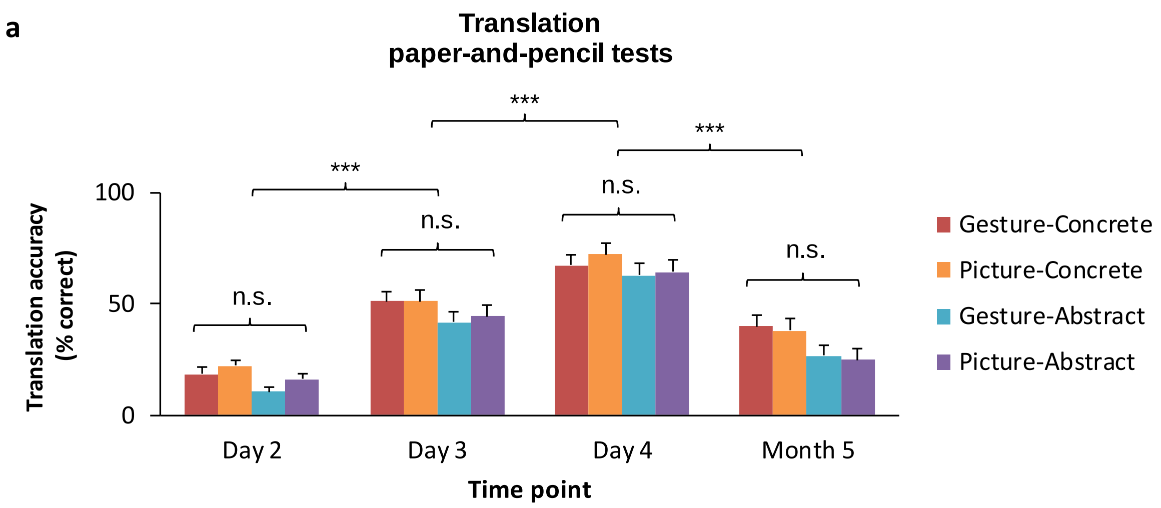

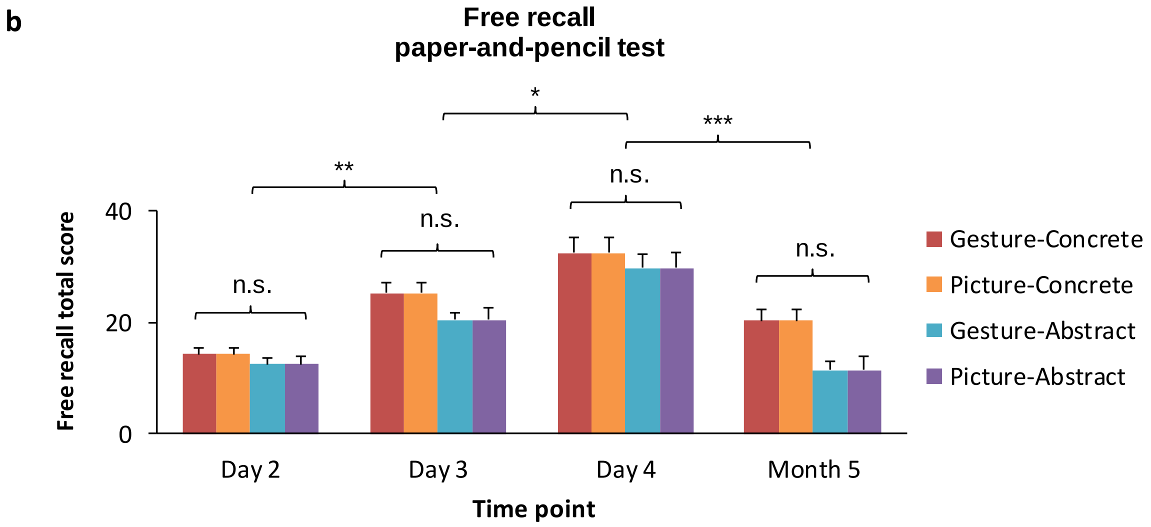

Figure S1. Paper-and-pencil vocabulary test scores. a, Performance on paper-and-pencil translation tests significantly improved during days 2 to 4 of gesture- and picture-enriched training. Performance declined 5 months following both gesture- and picture-enriched training (n = 22 participants). **b,** The same pattern of performance was observed for the free recall test (n = 22 participants). Error bars represent one standard error of the mean. *$p < .05$, **$p < .01$, ***$p < .001$.

Free recall test. We next examined performance on the free recall paper-and-pencil test. Points for correctly recalled German words, Vimmi words, and German-Vimmi translations were

summed for each participant, learning condition, testing time point, and vocabulary type (cf. Mayer et al., 2015). Free recall test scores by condition are shown in **Fig. S1b**. A three-way ANOVA on free recall scores with factors learning condition (gesture, picture), testing time point (day 2, day 3, day 4, month 5), and vocabulary type (concrete, abstract) did not yield any significant interactions of the learning condition factor with other variables besides a significant learning condition × vocabulary type interaction ($F_{1, 21}$ = 7.11, p = .014, two-tailed, $\eta_p^2 = .25$). Tukey's HSD post-hoc tests revealed higher scores for concrete words compared to abstract words following gesture-enriched learning but not picture-enriched learning (p < .001, Hedge's g = .40). There was a significant main effect of vocabulary type ($F_{1, 21}$ = 11.14, p = .003, two-tailed, $\eta_p^2 = .35$); scores were significantly higher for concrete words than abstract words. There was also a significant main effect of testing time point ($F_{3, 63}$ = 66.48, p < .001, two-tailed, $\eta_p^2 = .76$). Tukey's HSD post-hoc tests revealed that overall test scores were significantly higher at day 3 compared to day 2 (p = .0062, Hedge's g = 1.33), day 4 compared to day 3 (p = .014, Hedge's g = .85), and at month 5 compared to day 4 (p < .001, Hedge's g = 1.39). There was also a significant time point × vocabulary type interaction ($F_{3, 63}$ = 18.90, p < .001, two-tailed, $\eta_p^2 = .47$). There were no other significant main effects or interactions.

Sensorimotor-enriched training reduces long-term decrease in translation accuracy on paper-and-pencil vocabulary tests. Finally, we tested whether gesture-enriched learning diminished long-term decreases in translation accuracy over time compared to picture-enriched learning on the paper-and-pencil vocabulary tests.

Translation tests. In order to evaluate long-term changes in translation test accuracy, we computed the difference in mean performance on the translation tests (percent correct) at day 4 and month 5 for each participant, learning condition, and word type. A two-way ANOVA on difference scores (percent correct) with the factors learning condition (gesture, picture) and vocabulary type (concrete, abstract) yielded a significant main effect of learning condition ($F_{1, 21}$ =

5.84, p = .025, two-tailed, η_p^2 = .22). Performance decreased significantly less for gesture-enriched vocabulary compared to picture-enriched vocabulary 5 months following training (**Fig. S2**). There was also a significant main effect of vocabulary type ($F_{1, 21}$ = 8.75, p = .007, two-tailed, η_p^2 = .29). Performance decreased significantly less for concrete vocabulary compared to abstract vocabulary 5 months following training. The interaction between learning condition and vocabulary type variables was not significant.

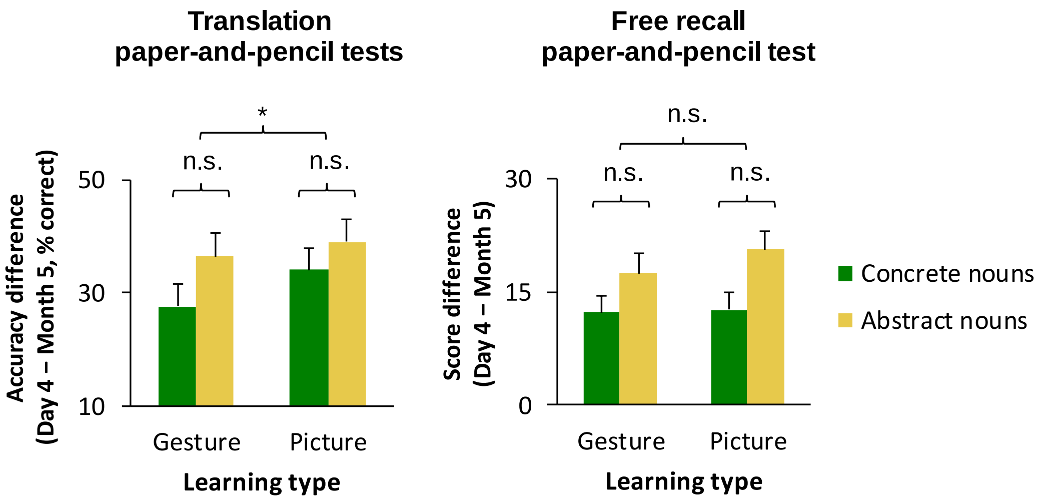

Figure S2. Long-term decrease in translation accuracy on paper-and-pencil vocabulary tests. Left: Gesture-enriched L2 vocabulary learning resulted in less of a decrease in performance on paper-and-pencil translation tests 5 months following learning compared to picture-enriched learning. Right: On the free recall paper-and-pencil test, participants demonstrated less long-term decay of concrete vocabulary compared to abstract vocabulary over a 5-month period (n = 22 participants). Error bars represent one standard error of the mean. *p < .05, **p < .01, ***p < .001.

Free recall test. In order to evaluate long-term changes in recall accuracy, we computed the difference in free recall paper-and-pencil test scores at day 4 and month 5 for each participant, learning condition, and word type. A two-way ANOVA on difference scores with the factors learning

condition (gesture, picture) and vocabulary type (concrete, abstract) yielded only a significant main effect of vocabulary type ($F_{1, 21}$ = 18.43, p < .001, two-tailed, $\eta_p^2 = .47$). Recall accuracy decreased significantly less for concrete vocabulary compared to abstract vocabulary 5 months following training (**Fig. S2**). The main effect of learning condition and interaction between learning condition and vocabulary type variables were not significant.

Summary. Taken together, the paper-and-pencil test scores revealed significant improvement for both gesture- and picture-enriched words from day 2 to day 3 and day 3 to day 4 of the L2 training period, as well as a significant decrease in performance 5 months post-learning compared to day 4. This pattern of performance was consistent across test types (translation and free recall tests). Analyses of L2 memory decay over a 5-month interval (day 4 scores – month 5 scores) revealed greater decay of memories for picture-enriched words compared to gesture-enriched words over a 5-month period on the translation tests. However, no difference between gesture- and picture-enriched words in terms of amount of decay was observed on the free recall tests. Instead, the free recall tests were sensitive to word type: A greater amount of decay was observed for abstract words compared to concrete words based on free recall scores.

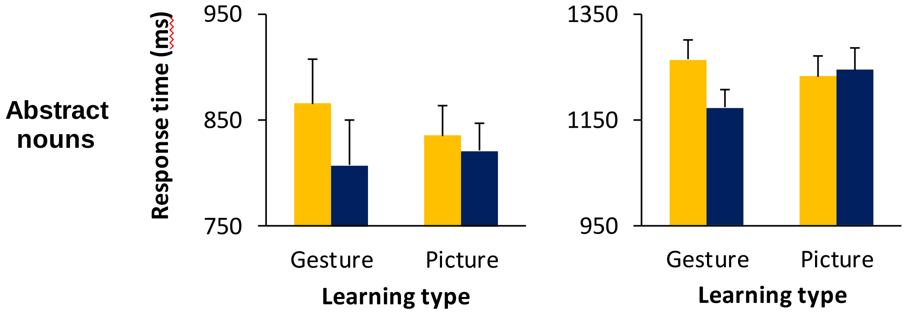

Figure S3. Post-hoc control analysis examining effects of bmSTS stimulation on the translation of abstract words. Response times in the multiple choice task are shown for only abstract L2 words that were translated correctly by each participant at both of the testing time

points. Responses are shown for the day 5 TMS session (left) and month 5 TMS session (right) by stimulation type and learning type (*n* = 22 participants). Overall, words learned with gesture enrichment were translated more slowly when TMS was applied to the bmSTS relative to sham stimulation ($F_{1,21}$ = 4.58, *p* = .044, two-tailed, $\eta_p^2 =$ .18; **Fig. S3**; see **Table S9** for the full set of ANOVA results). There were no significant two-way interactions and the three-way interaction was not significant (all *p*s > .40).

Analysis of variance (ANOVA) summary tables

In this section, we summarize using tables the full set of main effects and interactions for all ANOVA analyses reported in the main manuscript. *p < .05, **p < .01, ***p < .001.

Table S4. Two-way ANOVA testing effects of stimulation type and learning condition on response times in the multiple choice translation task.

	df	*F*	*p*	η_p^2
Intercept	21	927.96	<.001	
Stimulation	21	2.49	.13	.11
Learning	21	.003	.96	<.001
Stimulation × Learning	21	11.82	.002**	.36

Table S5. Three-way ANOVA testing effects of stimulation type, learning condition, and time point on response times in the multiple choice translation task.

	df	*F*	*p*	η_p^2
Intercept	21	1013.03	<.001	
Stimulation	21	3.30	.084	.14
Learning	21	1.18	.29	.05
Time	21	18.65	<.001***	.84

Stimulation × Learning	21	106.95	<.001***	.47
Stimulation × Time	21	1.23	.28	.06
Learning × Time	21	6.42	.019*	.23
Stimulation × Learning × Time	21	7.51	.012*	.26

Table S6. Two-way ANOVA testing effects of stimulation type and learning condition on response times in the multiple choice translation task at month 5.

	df	*F*	*p*	η_p^2
Intercept	21	1377.78	<.001	
Stimulation	21	2.94	.10	.12
Learning	21	3.81	.07	.15
Stimulation × Learning	21	21.81	<.001***	.51

Table S7. Two-way ANOVA testing effects of stimulation type and learning condition on response times in the multiple choice translation task at day 5.

	df	*F*	*p*	η_p^2
Intercept	21	510.00	<.001	
Stimulation	21	2.31	.14	.10
Learning	21	1.82	.19	.08
Stimulation × Learning	21	1.00	.33	.05

Table S8. Four-way ANOVA testing effects of stimulation type, learning condition, time point, and vocabulary type on response times in the multiple choice translation task.

	df	*F*	*p*	η_p^2
Intercept	21	1055.04	<.001	
Stimulation	21	2.07	.17	.09
Learning	21	.54	.54	.02
Time	21	131.51	<.001***	.86
Vocabulary	21	25.48	<.001***	.55
Stimulation × Learning	21	9.03	.007**	.30
Stimulation × Time	21	.014	.91	<.001
Learning × Time	21	8.29	.009**	.28
Stimulation × Vocabulary	21	.25	.62	.01
Learning × Vocabulary	21	.03	.86	.001
Time × Vocabulary	21	3.30	.083	.14
Stimulation × Learning × Time	21	10.97	.003**	.34
Stimulation × Learning × Vocabulary	21	2.95	.10	.12
Stimulation × Time × Vocabulary	21	.44	.52	.02
Learning × Time × Vocabulary	21	.11	.74	.005
Stimulation × Learning × Time × Vocabulary	21	5.24	.033*	.20

Table S9. Three-way ANOVA testing effects of stimulation type, learning condition, and time point on response times in the multiple choice translation task, restricted to only L2 words that were translated correctly at both testing time points.

	df	*F*	*p*	η_p^2
Intercept	21	2203.81	<.001	
Stimulation	21	2.32	.14	.10
Learning	21	.06	.81	.003
Time	21	241.23	<.001***	.92
Stimulation × Learning	21	4.58	.044*	.18

Stimulation × Time	21	.004	.95	<.001
Learning × Time	21	.20	.66	.01
Stimulation × Learning × Time	21	.75	.40	.03

Table S10. Four-way ANOVA testing effects of stimulation type, learning condition, time point, and vocabulary type on accuracy in the multiple choice translation task.

	df	*F*	*p*	η_p^2
Intercept	21	361.33	<.001	
Stimulation	21	1.13	.30	.05
Learning	21	.001	.97	<.001
Time	21	124.77	<.001***	.86
Vocabulary	21	35.62	<.001***	.63
Stimulation × Learning	21	.035	.85	.002
Stimulation × Time	21	.19	.67	.009
Learning × Time	21	6.86	.016*	.25
Stimulation × Vocabulary	21	3.86	.06	.15
Learning × Vocabulary	21	1.25	.28	.06
Time × Vocabulary	21	3.93	.90	.16
Stimulation × Learning × Time	21	.016	.90	<.001
Stimulation × Learning × Vocabulary	21	3.54	.074	.14
Stimulation × Time × Vocabulary	21	.013	.91	<.001
Learning × Time × Vocabulary	21	2.60	.12	.11
Stimulation × Learning × Time × Vocabulary	21	8.23	.009**	.28

Supplementary References

Bates D, Maechler M, Bolker B, Walker S. (2015). Fitting linear mixed-effects models using lme4. J Stat Softw. 67:1-48.

Köper M, Schulte im Walde S. 2016. Automatically generated affective norms of abstractness, arousal, imageability and valence for 350,000 german lemmas. Proc Int Conf Language Resources Evaluation. 2595-2598.

Kuznetsova A, Brockhoff PB, Christensen RHB. 2017. lmerTest package: Tests in linear mixed effects models. J Stat Softw. 82:1-26.

Lenth R, Singmann H, Love J, Buerkner P, Herve M. 2019. Package "emmeans": Estimated Marginal Means, aka Least-Squares Means. Compr R Arch Netw. 1-67.

Mayer KM, Yildiz IB, Macedonia M, von Kriegstein K. 2015. Visual and motor cortices differentially support the translation of foreign language words. Curr Biol. 25:530-535.
